# Human iPSC‐NSC‐Derived Extracellular Vesicles Can Alleviate Alzheimer's Disease‐Linked Impairments in Mitochondria, mTOR Signaling, Autophagy, and Hippocampal Neurogenesis

**DOI:** 10.1111/acel.70590

**Published:** 2026-06-16

**Authors:** Leelavathi N. Madhu, Sahithi Attaluri, Sanya Kotian, Raghavendra Upadhya, Yogish Somayaji, Shama Rao, Prashant Tarale, Shruthi V. Ganesh, Charles Huard, Maheedhar Kodali, Bing Shuai, Vidya V. Rao, Ashok K. Shetty

**Affiliations:** ^1^ Institute for Regenerative Medicine, Department of Cell Biology and Genetics Texas A&M University Naresh K Vashisht College of Medicine Bryan/College Station Texas USA

**Keywords:** Alzheimer's disease, autophagy, hippocampal neurogenesis, mitochondrial dysfunction, Mitophagy, mTOR signaling

## Abstract

Intranasal (IN) administrations of extracellular vesicles (EVs) derived from human‐induced pluripotent stem cell (hiPSC)‐derived neural stem cells (hNSCs) have shown promise in reducing chronic neuroinflammation mediated by microglia and astrocytes in 5x familial Alzheimer's disease (5xFAD) mice, a model for early‐onset Alzheimer's disease (AD). The current study rigorously investigated whether treatment with hiPSC‐NSC‐EVs could also alleviate several other neuropathological changes contributing to progressive cognitive decline. Three‐month‐old male and female 5xFAD mice received IN administrations of either hiPSC‐NSC‐EVs (~30 × 10^9^/week for 2 weeks) or vehicle. Two months later, the hippocampus of both male and female 5xFAD mice treated with the vehicle showed increased levels of markers of oxidative stress and mechanistic target of rapamycin (mTOR) signaling, altered expression of genes and/or proteins linked to mitochondria and autophagy, and diminished neurogenesis. In contrast, treatment with hiPSC‐NSC‐EVs restored levels of oxidative stress markers and the expression of genes and/or proteins linked to various mitochondrial complexes, mitochondrial biogenesis, fission, fusion, and mitophagy closer to naïve control levels, indicating alleviation of mitochondrial impairments. These improvements were accompanied by reduced phosphorylated mTOR levels and multiple autophagy markers matching those in naïve controls, suggesting a dampening of mTOR signaling and an enhancement of autophagy. Furthermore, mice treated with hiPSC‐NSC‐EVs showed increased hippocampal neurogenesis, associated with enhanced brain‐derived neurotrophic factor signaling. Overall, the results highlight that IN administrations of hiPSC‐NSC‐EVs in the early stages of AD can help slow the progression of multiple neuropathological changes associated with cognitive decline in 5xFAD mice and potentially AD.

## Graphical abstract

Intranasal administration of extracellular vesicles derived from human‐induced pluripotent stem cell‐derived neural stem cells in 5xFAD mice results in reduced oxidative stress, increased expression of proteins and genes associated with mitochondrial biogenesis and electron transport chain function, decreased mTOR activation, enhanced autophagy and mitophagy, and elevated hippocampal neurogenesis.

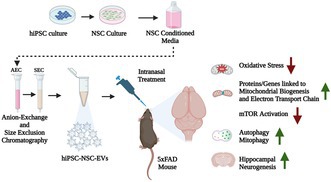

## 1 Introduction

Alzheimer's disease (AD), a progressive neurodegenerative disorder, is the leading cause of dementia worldwide (Masurkar et al. 2024). The gradual decline of cognitive functions, including memory and reasoning, is a hallmark of AD (Self and Holtzman 2023). AD primarily manifests through the accumulation of extracellular amyloid beta (Aβ) plaques and phosphorylated tau tangles, also known as neurofibrillary tangles (NFTs), within neurons in the brain, leading to synapse loss, neurodegeneration, and cognitive decline (Arnsten et al. 2025). Currently, two monoclonal antibodies targeting Aβ are being used as a breakthrough strategy to modify the progression of AD (Paczynski et al. 2025). However, the long‐term effectiveness of these antibodies in restraining the progression of AD pathogenesis is still uncertain (Raket et al. 2024), as they do not directly address other AD‐related pathologies such as enhanced oxidative stress, neuroinflammation, mitochondrial dysfunction, hyperactivation of mechanistic target of rapamycin (mTOR), and declined autophagy, hippocampal neurogenesis, and brain‐derived neurotrophic factor (BDNF) signaling.

Incessant oxidative stress is one of the brain alterations contributing to progressive cognitive impairments in AD, as elevated levels of reactive oxygen species (ROS) can increase the production of Aβ (Ionescu‐Tucker and Cotman 2021) and promote the formation of NFTs by increasing tau phosphorylation (Melov et al. 2007). Elevated oxidative stress also causes mitochondrial dysfunction, which could involve several alterations in mitochondria, including the downregulation of peroxisome proliferator‐activated receptor‐γ coactivator‐1α (PGC‐1α), the primary regulator of mitochondrial biogenesis, altered mitochondrial fission and fusion dynamics, and impaired mitophagy (Sheng et al. 2012; Wang et al. 2020). Neurons, like other cells in the body, maintain a healthy pool of mitochondria through continuous fission and fusion mediated by mitochondrial fission proteins, and impairments in either fission or fusion cause mitochondrial dysfunction (Mishra and Chan 2014). Furthermore, mitochondria are highly susceptible to damage due to the considerable production of ROS while performing their function; hence, they have a built‐in quality control system called mitophagy, which degrades damaged mitochondria. Mitophagy involves stabilization or activation of PTEN‐induced putative kinase 1 (PINK1) at the outer mitochondrial membrane by impaired mitochondrial membrane potential, denoting damaged mitochondria (Nguyen et al. 2016). PINK1 then phosphorylates and recruits the E3‐ubiquitin ligase, PARKIN, to mitochondria and phosphorylates ubiquitin to feed PARKIN‐mediated ubiquitination of mitochondrial outer membrane proteins for degradation through mitophagy (Wang et al. 2020; Fischer et al. 2012). Studies in animal models have implied that AD is associated with mitochondrial dysfunction associated with impaired biogenesis and mitophagy (McGill Percy et al. 2025; D'Alessandro et al. 2025).

Furthermore, damaged proteins and organelles accumulate when the mTOR signaling is activated (Perluigi et al. 2021). In AD, mTOR hyperactivation substantially diminishes autophagy by phosphorylating proteins involved in autophagosome formation, resulting in reduced clearance of Aβ and NFTs (Perluigi et al. 2021). Moreover, hippocampal neurogenesis in the adult brain adds newly born neurons to the existing adult hippocampal circuitry (Kempermann and Gage 2000; Rao and Shetty 2004; Gonçalves et al. 2016), which also diminishes sharply in the early stages of AD, and the decline in hippocampal neurogenesis correlates with the cognitive impairments in AD patients (Salta et al. 2023; Zanirati et al. 2023). Hippocampal neurogenesis also declines rapidly in 5xFAD mice, an animal model of early onset AD (Choi et al. 2018). Additionally, the maintenance of the BDNF, the extracellular signal‐regulated kinase (ERK), and cAMP response element‐binding protein (CREB) signaling pathway in the hippocampus regulates the extent of new neuron addition to the hippocampal circuitry (Jiang et al. 2015; Kodali et al. 2023a). Also, BDNF concentration wanes significantly in the hippocampus of AD patients and in 5xFAD mice (Choi et al. 2018; Gao et al. 2022), likely due to Aβ‐induced inhibition of CREB phosphorylation (Vitolo et al. 2002). Therefore, in addition to treatments that promote amyloid clearance and reduce tau phosphorylation, strategies that alleviate oxidative stress, neuroinflammation, mitochondrial dysfunction, mTOR activation, and declines in autophagy, hippocampal neurogenesis, and BDNF signaling will help slow the progression of AD.

A recent study from our laboratory investigated the effects of intranasal (IN) administration of extracellular vesicles (EVs) from human induced pluripotent stem cell (hiPSC)‐derived neural stem cells (NSCs) in 5x familial Alzheimer's disease (5xFAD) mice, a model of early‐onset AD (Madhu et al. 2024). Treatment with hiPSC‐NSC‐EVs that are naturally enriched with multiple miRNAs capable of mediating antioxidant, anti‐inflammatory, neuroprotective, and neurogenic effects (Upadhya et al. 2020, 2022; Rao et al. 2025) modulated the transcriptome of activated microglia and astrocytes at the single‐cell level in 5xFAD mice. The treatment also reduced the activation of several neuroinflammatory signaling cascades in male and female 5xFAD mice, including the nucleotide‐binding oligomerization domain (NLR) family pyrin domain‐containing 3 (NLRP3) and the downstream p38 mitogen‐activated protein kinase (MAPK) pathway, as well as the interferon‐1 (IFN‐1) signaling pathway. Notably, the anti‐inflammatory effects were also associated with reductions in amyloid plaque load and tau phosphorylation (Madhu et al. 2024). hiPSC‐NSC‐EVs treatment also maintained better object location memory, a hippocampus‐dependent cognitive function, and pattern separation ability, a task dependent on the integrity of the dentate gyrus and the extent of hippocampal neurogenesis, for extended periods in both sexes (Madhu et al. 2024). However, improved hippocampus‐dependent cognitive functions in AD models could be influenced by multiple factors beyond neuroinflammation, such as improved mitochondrial function (Gupta et al. 2025), reduced mTOR activation (Gourmaud et al. 2022), activation of autophagy and mitophagy (Hou et al. 2024), and improved hippocampal neurogenesis and BDNF signaling (Choi et al. 2018). Therefore, in this study, by employing brain tissues from male and female naïve mice, and male and female 5xFAD mice that received IN administrations of hiPSC‐NSC‐EVs or Vehicle (Veh), which have already been tested for cognitive and memory function in our previous study (Madhu et al. 2024), we explored the other factors that could contribute to improved cognitive function. Particularly, we evaluated the effects of hiPSC‐NSC‐EVs treatment in 5xFAD mice on (a) markers of oxidative stress, (b) genes and/or proteins linked to mitochondrial function, mTOR activation, and autophagy; (c) hippocampal neurogenesis, and (d) brain‐derived neurotropic factor (BDNF) signaling, in comparison to Veh treatment.

## 2 Materials and Methods

### 2.1 Production and Characterization of hiPSC‐NSC‐EVs

Our previous reports have provided detailed descriptions of the steps involved in the production and characterization of hiPSC‐NSC‐EVs (Madhu et al. 2024; Upadhya et al. 2020, 2022). These include the methods for generating NSCs from hiPSCs using the IMR90‐4 cell line obtained from the Wisconsin International Stem Cell Bank (Madison, WI, USA), culturing hiPSC‐NSCs, isolating hiPSC‐NSC‐EVs through anion‐exchange and size‐exclusion chromatographic methods, and characterizing for various EV‐specific markers and ultrastructural features, as per MISEV2023 guidelines (Welsh et al. 2024). Our previous small RNA sequencing studies have demonstrated the presence of miR‐122‐5p, miR‐99b‐5p, and miR‐16‐5p in hiPSC‐NSC‐EVs, in addition to multiple other miRNAs (Upadhya et al. 2020). Because of their proficiency in suppressing mTOR signaling, we confirmed their expression in hiPSC‐NSC‐EVs by quantitative real‐time polymerase chain reaction (qRT‐PCR). U6snRNA was used as a housekeeping gene to normalize the miRNA levels. The qRT‐PCR methods employed for measuring miRNAs are available in our previous report (Rao et al. 2025).

### 2.2 Animals, Study Design, and Intranasal Administration of hiPSC‐NSC‐EVs

**FIGURE 1:**
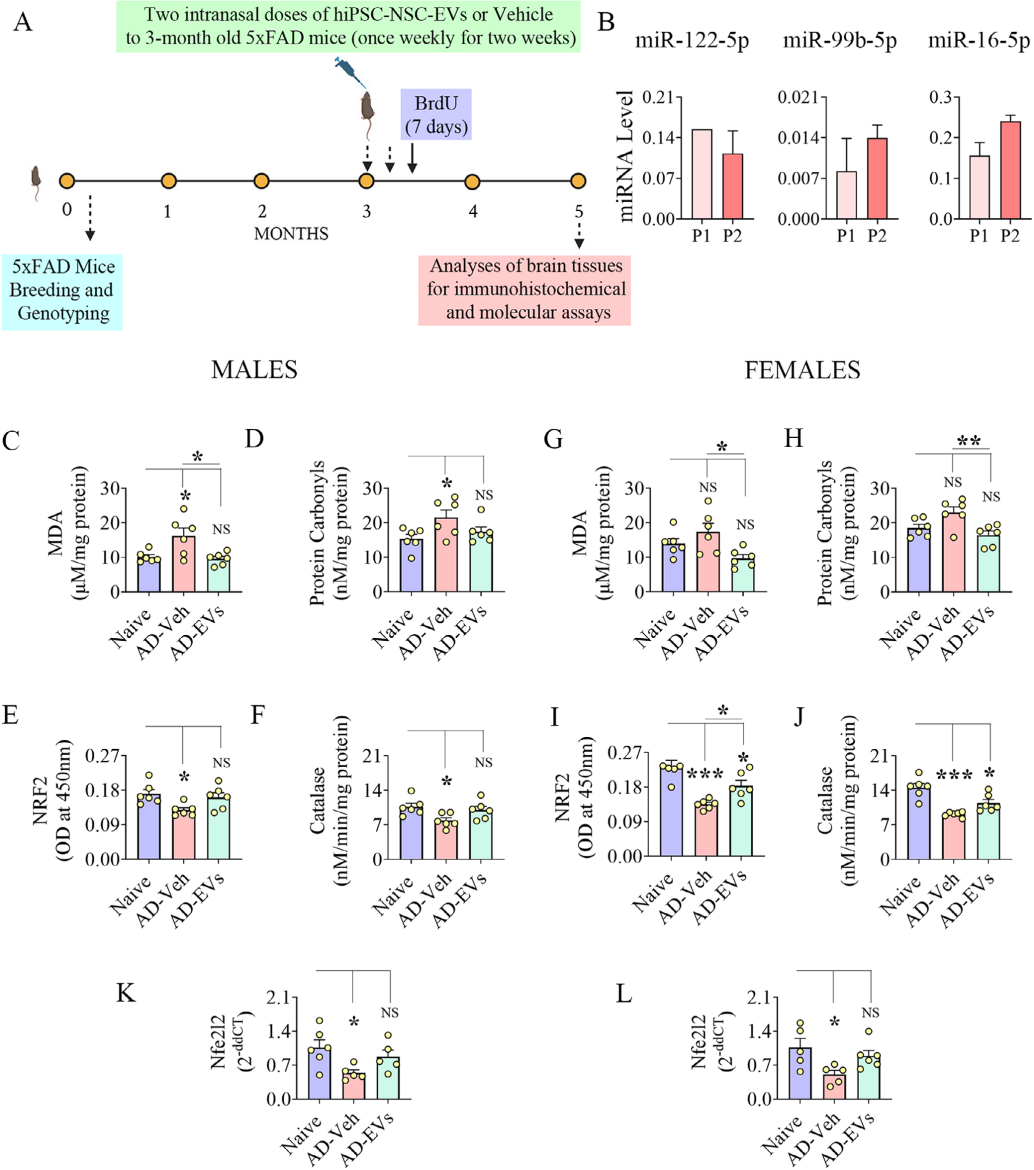
The cartoon in (A) illustrates the overview of the research design. The timelines for human‐induced pluripotent stem cell‐derived neural stem cells (hiPSC‐NSC‐EVs) treatment, 5′‐bromodeoxyuridine (BrdU) injections, and brain tissue analyzes in 5xFAD mice are shown. Three‐month‐old male and female 5xFAD mice received two doses of 30 × 10^9^ hiPSC‐NSC‐EVs or vehicle (phosphate‐buffered saline), once weekly for 2 weeks. A week after the second dose, the animals designated for histological studies received BrdU (100 mg/Kg, i.p., once daily for 7 days) to facilitate quantification of hippocampal neurogenesis. All animals were euthanized at 5 months old, and brain tissues were processed for immunohistochemical and molecular assays. Bar chart (B) displays levels of miR‐122‐5p, miR‐99b‐5p, and miR‐16‐5p in two biological replicates of hiPSC‐NSC‐EVs preparations. Bar graphs (C–J) show the levels of malondialdehyde; MDA (C, G), protein carbonyls (D, H), nuclear factor erythroid‐2; NRF2 (E, I), catalase (F, J), and Nfe2l2 (K, L) in males (C‐F, K) and females (G‐J, L) from different groups. *p < 0.05, **p < 0.01, and ***p < 0.001; NS, not significant.

The current study employed brain tissues harvested for our recently published study, which focused on the beneficial effects of IN administration of hiPSC‐NSC‐EVs on microglia, astrocytes, neuroinflammation, and cognitive function in 5xFAD mice (Madhu et al. 2024). The study comprised three groups of animals, comprising both sexes. These include 5xFAD mice (Cat No: 34840‐JAX, Bar Harbor, Maine, USA) receiving Veh (AD‐Veh group, *n* = 26, 14 males and 12 females) or hiPSC‐NSC‐EVs treatment (AD‐EVs group, *n* = 27, 13 males and 14 females) and age‐matched wild type mice from the same background (B6SJLF1/J) as the 5xFAD mice (Naive group, *n* = 24, 12 males and 12 females). The sequence of experiments in the previously published study (Madhu et al. 2024) was as follows: (a) 5xFAD mice in the AD‐EVs group received two intranasal doses of 30 × 10^9^ EVs, whereas their counterparts in the AD‐Veh group received phosphate‐buffered saline (PBS), with a 1‐week interval between doses when they were 3 months old (Figure 1A). (b) A week after the second dose, subgroups of mice designated for histological studies in all groups received injections of 5′‐bromodeoxyuridine (BrdU, 100 mg/Kg, once daily for 7 days) to facilitate the quantification of net hippocampal neurogenesis. (c) The behavioral tests commenced 3 weeks after Veh/EVs administration which required a month to complete because of multiple tests (i.e., until the 5th month of life). (d) Immediately after the completion of behavioral tests, animals were euthanized and brain tissues harvested (when they were 5 months old). Thus, animals underwent behavioral tests for a month (i.e., during their 4th and 5th month of life, equivalent to 1–2 months post‐EVs administration) before undergoing euthanasia. Since pathogenesis in the brain of 5xFAD mice progresses rapidly, with considerable amyloid deposits and neuroinflammation, we chose to test a relatively higher dose of hiPSC‐NSC‐EVs (i.e., 30 × 10^9^ EVs, once weekly for 2 weeks) compared to lower doses of hiPSC‐NSC‐EVs (∼10 × 10^9^ EVs) employed in our previous study in a mouse model of peripheral inflammation‐induced cognitive dysfunction (Ayyubova et al. 2023). The detailed method for intranasal administration of hiPSC‐NSC‐EVs is described in our previous report (Madhu et al. 2024). The Institutional Animal Care and Use Committee of Texas A&M University approved all animal experiments performed in this study.

### 2.3 Harvesting of Brain Tissues, and Preparation of Lysates From Hippocampal Tissues

The brain tissue sections for immunohistochemical analyzes in this study were from subgroups of animals from all groups (*n* = 5–6/group) that underwent intracardiac perfusions with 4% paraformaldehyde and serial coronal cryostat sectioning of brains at 30‐μm thickness (Madhu et al. 2024). The brain tissues used for biochemical and molecular biological analyzes in this study were obtained from subgroups of animals from all groups (*n* = 5–6/group) undergoing fresh brain harvesting following deep anesthesia and decapitation (Madhu et al. 2024). The fresh brain tissues were snap‐frozen in liquid nitrogen and stored at −80°C. The hippocampi were first micro‐dissected from the harvested fresh brains and were individually lysed through sonication in a tissue extraction reagent (Invitrogen, Waltham, MA, USA) containing protease inhibitor (Sigma Aldrich, St. Louis, MO, USA; 1:100 dilution) for 15–20 s at 4°C. The resulting solutions were centrifuged for 10 min at 13000 rpm, and the supernatants were aliquoted and stored at −80°C until further use. The methods for harvesting fixed and fresh tissues and preparation of brain tissue lysates were detailed in our previous reports (Shetty et al. 2020; Madhu et al. 2019, 2021; Kodali et al. 2023b).

### 2.4 Measurement of Oxidative Stress Markers

Commercially available kits were used to measure oxidative stress in hippocampal lysates. The markers included malondialdehyde (MDA, Cayman Chemicals, Arbor, MI, USA), protein carbonyls (PC, Cayman Chemicals), and the transcription factor nuclear factor erythroid 2‐related factor 2 (NRF2, Signosis, Santa Clara, CA, USA). The NRF2 ELISA kit used in this study provides high sensitivity for detecting activated NRF2. Each 96‐well plate is pre‐coated with an NRF2 consensus sequence oligonucleotide. Upon addition of cell lysate, activated NRF2 binds specifically to this oligonucleotide. Detection is subsequently accomplished using a primary antibody specific to an NRF2 subunit, followed by an HRP‐conjugated secondary antibody. Additionally, the antioxidant catalase (CAT, Cayman Chemicals) was measured. We followed the manufacturer's instructions for all assays. We utilized a Pierce BCA reagent kit to assess the total protein content in the various tissue lysates (ThermoFisher Scientific, Waltham, MA, USA). The concentrations of the markers were then normalized to 1 mg of total protein in the corresponding tissue lysates. Furthermore, we measured Nfe2l2 (NRF2) levels by qRT‐PCR. Briefly, RNA was isolated from hippocampal tissues using a kit from Qiagen (Germantown, MD, USA). The isolated samples, with a concentration of 500 ng/μL, were converted into complementary DNA (cDNA) using the RT^2^ First Strand Kit from Qiagen. qRT‐PCR was performed using the RT^2^ SYBR Green qPCR Mastermix and Primer mix from GeneCopoeia (Rockville, MD, USA) to measure the expression of Nfe2l2. Gapdh was used as a housekeeping gene to normalize the gene expression levels. 2^−ddCT^ values were calculated compared to the naïve group.

### 2.5 Measurement of the Expression of Mitochondrial Respiratory Chain Genes

The cDNA samples were used to assess the expression of select mitochondrial respiratory chain genes: Ndufs6, Ndufs7, Sdha, Sdhb, Cyc1, Bcs1l, Cox7b, Cox4i2, Slc25a1, and Atp6ap1. Gapdh served as the housekeeping gene for normalization, and 2^−ddCT^ values were calculated relative to the naïve group, as previously described (Madhu et al. 2024). Mitochondrial DNA‐encoded genes (Mt‐Nd1, Mt‐Co1, and Mt‐Atp8) were quantified using the QuantiNova SYBR Green PCR Kit and QuantiNova LNA Primer mix (Qiagen), with 18 s rRNA as the normalization control. Gene expression was expressed as 2^−ddCT^ values compared to the naïve group.

### 2.6 Mitochondrial Function Assay

To understand mitochondrial function, we measured the activities of succinate dehydrogenase (SDH) and the levels of NAD+/NADH and ATP in the naïve, AD‐Veh, and AD‐EVs groups. We followed the manufacturer's protocol (Signosis, Santa Clara, CA, USA). All assay measurements were normalized to 1 mg of total protein in all hippocampal lysates of the study group. The detailed protocol of these assays is provided in the supplementary file.

### 2.7 Quantification of Markers for Mitochondrial Biogenesis and Mitophagy

Hippocampal lysates were analyzed to determine the concentrations of key markers of mitochondrial biogenesis and mitophagy (*n* = 5–6 per group) using commercial ELISA kits as previously described (Madhu et al. 2021, 2024; Kodali et al. 2023b). Kits for PGC‐1α (Aviva Systems Biology, San Diego, CA, USA), Fis1 (Abbexa, Sugar Land, TX, USA), Mfn1 (MyBioSource, San Diego, CA, USA), PINK1 (Abbexa, Sugar Land, TX, USA), and PARKIN (MyBioSource) were used. Protein concentrations were normalized to 1 mg of total hippocampal protein and compared between groups. In addition, PINK1 and PARKIN gene expression was assessed by qRT‐PCR, with Gapdh as the housekeeping gene. The expression levels (2^−ddCT^) were calculated relative to the naïve group. Furthermore, Western blotting was performed for DRP1, involved in mitochondrial fission, and OPA1, a central regulator of mitochondrial fusion, with detailed methods provided in the supplementary file.

### 2.8 Quantification of Proteins Linked to mTOR Signaling and Autophagy

The hippocampal lysates were processed to quantify the concentrations of pan‐mTOR, phosphorylated mTOR (p‐mTOR), and various autophagy‐related proteins, including beclin‐1, autophagy‐related gene 5 (ATG‐5), and microtubule‐associated protein 1 light chain 3B (MAP1LC3‐II, referred to as LC3‐II here onwards), using commercially available ELISA kits. The kits for pan‐mTOR and p‐mTOR were from Ray Biotech (Peachtree Corners, GA, USA), while beclin‐1 was obtained from Aviva Systems Biology. ATG‐5 and LC3‐II kits were from MyBioSource. We also measured Unc‐51‐like kinase 1 (ULK1) and ATG13 gene expression using qRT‐PCR. GAPDH was used as a housekeeping gene to normalize the gene expression levels. 2^−ddCT^ values were calculated by comparing to the naïve group. Additionally, we performed western blot analysis for LC3‐I and LC3‐II and calculated the LC3‐II/LC3‐I ratio. A detailed procedure for the Western blot is provided in the supplemental file.

### 2.9 Dual Immunofluorescence Methods for Visualizing Phosphorylated Ribosomal Protein S6 (pS6) and p62

Dual immunofluorescence procedures were used to visualize neurons or microglia co‐expressing ribosomal protein phosphorylated S6 (pS6, a marker of downstream mTOR signaling) and p62 (sequestosome 1, a marker of autophagy). After washing with PBS, brain tissue sections were blocked using 10% normal donkey serum and then incubated overnight at 4°C in a cocktail of two primary antibodies (pS6 and NeuN, pS6 and IBA‐1, or p62 and NeuN). The following day, the sections were treated with corresponding secondary antibodies. After rinsing in PBS, the sections were coverslipped with a slow fade/antifade mounting medium (Invitrogen). The primary antibodies used included chicken anti‐NeuN (1:1000, Abcam, Waltham, MA, USA) or rabbit anti‐IBA‐1 (1:1000, Abcam), rabbit anti‐phospho‐S6 (phosphorylation sites are Ser235/236, 1:200, Cell Signaling, Danvers, MA, USA), and guinea pig anti‐p62 (1:500, Progen, Heidelberg, Germany). The secondary antibodies consisted of donkey anti‐chicken IgG tagged with Alexa Fluor 405 (1:200, Abcam), donkey anti‐chicken IgG tagged with Alexa Fluor 488 (1:200, Invitrogen), donkey anti‐rabbit IgG tagged with Alexa Fluor 594 (1:200, Invitrogen), and donkey anti‐guinea pig IgG tagged with Alexa Fluor 594 (1:200, Jackson ImmunoResearch, West Grove, Pennsylvania, USA).

### 2.10 Measurement of the Extent of pS6 Expression in Hippocampal Neurons

The extent of pS6 expression was quantified within NeuN+ neurons in the hippocampal CA3 pyramidal neurons and IBA‐1+ microglia in the hippocampus using Z‐section analysis in a Leica THUNDER 3D Imager (3 images/section, three sections/animal, *n* = 5–6 animals/group) and ImageJ. A cropped image comprising the CA3 cell layer in each Z‐section was employed. We measured the fractions of pS6 expression in the soma of individual neurons in these images using ImageJ and the percentage of microglia expressing pS6 in the hippocampus.

### 2.11 Measurement of p62+ Structures in Hippocampal Neurons

We measured the fractions of p62+ structures in the hippocampal CA3 cell layer using Z‐section analysis in a Leica THUNDER 3D Imager (3 images/section, three sections/animal, *n* = 5 animals/group). For this analysis, a cropped image comprising the CA3 cell layer in each Z‐section was employed. The area fractions of p62 in the hippocampal CA3 cell layer were measured in each section using ImageJ.

### 2.12 BrdU and Doublecortin (DCX) Immunohistochemistry

Serial sections (every 15th) through the hippocampus were selected from each animal (*n* = 5–6 per group) and processed for single BrdU immunostaining using a monoclonal antibody specific to BrdU (rat anti‐BrdU, 1:500; Abcam). Another series of sections (every 15th) was processed for single DCX immunostaining using a polyclonal antibody against DCX (goat anti‐DCX, 1:300; Santa Cruz Biotechnology, Dallas, TX, USA). Our previous reports have detailed procedures for BrdU and DCX immunostaining (Rao and Shetty 2004; Rao et al. 2008; Hattiangady et al. 2008). In brief, the sections underwent several treatments for BrdU immunostaining. These included a 15‐min exposure to 20% methanol and 3% hydrogen peroxide at room temperature, followed by a thorough wash in Tris‐buffered saline (TBS). The sections were then incubated in a 50% formamide solution for 2 h at 65°C, treated with 2 N HCl for 60 min at 37°C, and placed in a 0.1 M borate buffer (pH 8.5) for 10 min at room temperature. After a 30‐min incubation with 10% blocking serum at room temperature, the sections were exposed to rat anti‐BrdU antibody overnight at 4°C. Next, the sections were washed in PBS and visualized for BrdU using a biotinylated anti‐rat IgG, the avidin‐biotin complex (ABC) reagent (Elite ABC kit; Vector labs, Burlingame, CA, USA), and diaminobenzidine (DAB, Vector labs) as the chromogen. For DCX immunostaining, the sections were treated with 10% blocking serum for 30 min at room temperature and then incubated overnight at 4°C with goat anti‐DCX. After washing in PBS, the sections were visualized for DCX using a biotinylated anti‐goat IgG, the ABC reagent, and Vector SG as the chromogen. The immunostained sections were mounted on gelatin‐coated slides, air‐dried, counterstained with hematoxylin (Vector Labs), dehydrated, cleared, and coverslipped.

### 2.13 Quantification of BrdU+ Newly Born Cells

To estimate the number of newly generated cells in the subgranular zone‐granule cell layer (SGZ‐GCL) of the dentate gyrus, every 15th section measuring 30 micrometers in thickness along the entire septo‐temporal axis of the hippocampus was processed for BrdU immunostaining and stereological counting. This analysis involved counting BrdU+ cells in the SGZ‐GCL across 5 sections per animal. The quantification process utilized a StereoInvestigator system (Microbrightfield, Williston, Vermont, USA) with a 100× objective lens, following the methodology detailed in our prior publications (Rao et al. 2008; Hattiangady et al. 2008).

### 2.14 Dual Immunofluorescence for Visualizing BrdU+ Newly Born Cells Differentiating Into Mature Neurons

The proportion of newly formed cells (i.e., BrdU+ cells) that differentiated into neurons was assessed through dual immunofluorescence for BrdU and NeuN, along with Z‐section analyzes performed on a Nikon confocal microscope, following the methods outlined in our earlier publications (Rao et al. 2008; Hattiangady et al. 2008). The antibodies that were employed include: rabbit anti‐NeuN (1:2000, Millipore Sigma, St. Louis, MO, USA), rat anti‐BrdU (1:500, Abcam), donkey anti‐rat IgG labeled with Alexa Fluor 594 (1:200, Invitrogen, Grand Island, NY, USA), and donkey anti‐rabbit IgG labeled with Alexa Fluor 488 (1:200, Invitrogen). Subsequently, the sections were washed in PBS and cover‐slipped with a slow fade/antifade mounting medium (Invitrogen). Optical Z‐sections, each measuring 2 μm in thickness, were utilized to calculate the percentages of BrdU+ cells that showed expression of NeuN. Following this, net hippocampal neurogenesis was determined by extrapolating the number of BrdU+ cells (newly born cells) in the SGZ‐GCL with the proportion of BrdU+ cells expressing the mature neuronal marker NeuN. This approach provided the number of new neurons incorporated into the hippocampal circuitry over a defined period, reflecting the net survival of neurons generated during the BrdU injection window.

### 2.15 Quantification of BDNF, p‐ERK and p‐CREB Proteins in the Hippocampus

The tissue lysates from the hippocampus were utilized to quantify the BDNF, p‐ERK (Thr202 and Tyr204), and p‐CREB (Ser133) proteins. Commercially available kits were used for measuring BDNF (ThermoFisher Scientific), p‐ERK1/2, and p‐CREB (Cell Signaling) following the manufacturer's instructions. The concentrations of these proteins were then normalized to 1 mg of total protein in the corresponding tissue lysates.

### 2.16 Statistical Analysis

The numbers of animals used for immunohistochemical and biochemical studies in each group were determined through a power analysis using G*Power software. We based the analysis on an effect size of 1.2 (derived from our previous data) and set the alpha level at 0.05. This analysis indicated that a minimum of 4 mice per group was necessary to achieve a power of 0.8 or higher. However, we utilized 5 to 6 mice per group to ensure robust datasets in all studies. We used GraphPad Prism (10.2) and employed one‐way ANOVA. For each parameter, we employed Tukey's multiple‐comparison post hoc tests after an ANOVA for 3 groups indicated significance to determine means from which specific groups differ. This limited the probability of making Type 1 errors (false positives). In cases where individual groups did not meet the assumptions of normality, we applied the Kruskal‐Wallis test, followed by Dunn's post hoc tests. A statistically significant *p*‐value of less than 0.05 was used for all comparisons.

## 3 Results

### 3.1 Characterization of hiPSC‐NSC‐EVs

The characterization of hiPSC‐NSC‐EVs employed in this study for EV‐specific markers and ultrastructure, as per MISEV2023 guidelines, has been described in detail in our recent publication (Madhu et al. 2024). Since the brain tissues of naïve mice and 5xFAD mice receiving hiPSC‐NSC‐EVs or Veh used for generating data in this article have come from animals reported in our previous publication (Madhu et al. 2024), the hiPSC‐NSC‐EVs characterization data are not repeated in this article but referred to the published article. However, in this study, we validated three miRNAs known to target the mTOR signaling pathway, which were identified in our previous small RNA sequencing analysis of hiPSC‐NSC‐EVs (27). These include miR‐122‐5p, miR‐99b‐5p, and miR‐16‐5p (Figure 1B). Furthermore, we have shown earlier that IN‐administered EVs incorporate into or interact with neurons and glia in the brain of 5xFAD mice (Attaluri et al. 2023). Examples of hiPSC‐NSC‐EVs incorporation into dentate granule cells, CA3 pyramidal neurons, and microglia in the hippocampus are illustrated in Figures S1 and S2.

### 3.2 hiPSC‐NSC‐EVs Treatment Reduced Oxidative Stress in the Hippocampus With NRF‐2 Upregulation

Male 5xFAD mice receiving Veh treatment (male AD‐Veh group) displayed increased concentrations of MDA and PCs compared to the naïve control group (*p* < 0.05, Figure 1C,D) reflecting the AD pathology. In contrast, in male 5xFAD mice receiving hiPSC‐NSC‐EVs (Male AD‐EVs group), both markers were near naïve control levels (*p* > 0.05, Figure 1C,D), with MDA concentration diminishing significantly below the male AD‐Veh group (*p* < 0.05, Figure 1C). The female AD‐Veh group also exhibited increased levels of MDA and PCs compared to the naïve group; however, the differences were not statistically significant (*p* > 0.05, Figure 1G,H). The concentrations of MDA and PCs in the female AD‐EVs group were significantly lower than those in the AD‐Veh group (*p* < 0.05–0.01, Figure 1G,H) and matched their levels in the female naïve group (*p* > 0.05, Figure 1G,H). Notably, reduced oxidative stress in the male AD‐EVs group was also associated with similar concentrations of NRF‐2 and CAT levels to those in the male naïve control group (*p* > 0.05, Figure 1E,F), in contrast to the male AD‐Veh group showing reduced concentrations of NRF‐2 and CAT compared to the male naïve control group (*p* < 0.05, Figure 1E,F). In the female AD‐EVs group, NRF‐2 concentration was increased compared to the female AD‐Veh group (*p* < 0.05, Figure 1I). The CAT concentration did not differ between female AD‐Veh and female AD‐EVs groups (*p* > 0.05, Figure 1J). We also measured Nfe2l2 gene expression in RNA samples isolated from hippocampal tissues across different study groups. In both male and female subjects, the level of Nfe2l2 was significantly reduced in the AD‐Veh group compared to the naïve group (*p* < 0.05, Figure 1K,L). However, in the hiPSC‐NSC‐EVs‐treated groups, these levels were similar to those in the naïve groups (*p* > 0.05, Figure 1K,L). Thus, hiPSC‐NSC‐EVs treatment diminished oxidative stress in the hippocampus by elevating NRF‐2 and catalase levels to those of naïve controls in male 5xFAD mice and increasing NRF‐2 concentration in female 5xFAD mice. The two‐way ANOVA analysis showed no differences based on sex or any interactions between sex and EVs treatment regarding MDA and PC levels and the expression of Nfe2l2. Nevertheless, the levels of NRF2 and CAT in naïve mice were significantly greater in females compared to males (Table 1).

**TABLE 1:** Analysis of sex differences using two‐way ANOVA for changes in markers of oxidative stress, mitochondrial health, mTOR signaling, autophagy, and hippocampal neurogenesis.

		Does sex affect the results?	Interaction between sex and treatment
Oxidative Stress	MDA	No (*p* > 0.05)	No (*p* > 0.05)
	PC	No (*p* > 0.05)	No (*p* > 0.05)
	NRF2	Yes (*p* < 0.01), Greater in females in naïve group (*p* < 0.0001)	No (*p* > 0.05)
	Catalase	Yes (*p* < 0.05), Greater in females in naïve group (*p* < 0.001)	No (*p* > 0.05)
	Nfe2l2	No (*p* > 0.05)	No (*p* > 0.05)
Mitochondrial Gene	Ndufs6	No (*p* > 0.05)	No (*p* > 0.05)
	Ndufs7	No (*p* > 0.05)	No (*p* > 0.05)
	Sdha	No (*p* > 0.05)	No (*p* > 0.05)
Biochemical examination qRT‐PCR	Sdhb	No (*p* > 0.05)	No (*p* > 0.05)
	Cyc1	No (*p* > 0.05)	No (*p* > 0.05)
	Bcs1l	No (*p* > 0.05)	No (*p* > 0.05)
	Cox7b	No (*p* > 0.05)	No (*p* > 0.05)
	Cox4i2	Yes (*p* < 0.05), Greater in males in AD‐EVs group (*p* < 0.05)	No (*p* > 0.05)
	Slc25a1	No (*p* > 0.05)	No (*p* > 0.05)
	Atp6ap1	No (*p* > 0.05)	No (*p* > 0.05)
Mitochondria encoded gene	MT‐CO1	No (*p* > 0.05)	Yes (*p* < 0.05), greater in males
	MT‐ND1	No (*p* > 0.05)	No (*p* > 0.05)
	MT‐ATP8	No (*p* > 0.05)	No (*p* > 0.05)
Mitochondrial Biogenesis	PGC1a	Yes (*p* < 0.05), Greater in females in naïve group (*p* < 0.01)	Yes (*p* < 0.05), but post hoc test implied similar effects in males and females
	Fis1	No (*p* > 0.05)	No (*p* > 0.05)
	Mfn1	Yes (*p* < 0.01), Greater in females in naïve group (*p* < 0.001)	Yes (*p* < 0.05), but post hoc test implied similar effects in males and females
	DRP1	No (*p* > 0.05)	No (*p* > 0.05)
	OPA1	No (*p* > 0.05)	Yes (*p* < 0.05), greater in males
	SDH activity	No (*p* > 0.05)	No (*p* > 0.05)
	NAD/NADH	No (*p* > 0.05)	No (*p* > 0.05)
	ATP	Yes (*p* < 0.05), Greater in females in naïve and AD‐Veh group (*p* < 0.01)	No (*p* > 0.05)
Mitophagy	PINK1	Yes (*p* < 0.0001), Greater in females in naïve and AD‐EVs groups (*p* < 0.01)	No (*p* > 0.05)
	PARKIN	No (*p* < 0.0001), Greater in females in naïve and AD‐EVs groups (*p* < 0.05–0.001)	No (*p* > 0.05)
qRT‐PCR	PINK1	No (*p* > 0.05)	No (*p* > 0.05)
	PRKN	No (*p* > 0.05)	No (*p* > 0.05)
mTOR	pan‐mTOR	Yes (*p* < 0.01) Greater in males of all groups (*p* < 0.05–0.0001)	No (*p* > 0.05)
	p‐mTOR	Yes (*p* < 0.01), Greater in males in naïve and AD‐EVs group (*p* < 0.05–0.01)	No (*p* > 0.05)
	p‐mTOR/pan‐mTOR Ratio	Yes (*p* < 0.001), Greater in females in AD‐Veh group (*p* < 0.0001)	Yes (*p* < 0.05), greater in females
	pS6 in Neuron	Yes (*p* < 0.001) Greater in females in all groups (*p* < 0.0001)	No (*p* > 0.05)
Autophagy	Beclin1	Yes (*p* < 0.05), Greater in females in AD‐Veh group (*p* < 0.05)	No (*p* > 0.05)
	ATG5	Yes (*p* < 0.001) Greater in females in naïve group (*p* < 0.0001)	Yes (*p* < 0.05), greater in males
	LC3‐II	Yes (*p* < 0.05) Greater in females in naive group (*p* < 0.05)	No (*p* > 0.05)
	ULK1	Yes (*p* < 0.05) Greater in males in AD‐EV group (*p* < 0.05)	No (*p* > 0.05)
	ATG13	No (*p* > 0.05)	No (*p* > 0.05)
	LC3‐I	No (*p* > 0.05)	No (*p* > 0.05)
	LC3‐II	No (*p* > 0.05)	No (*p* > 0.05)
	LC3‐II/LC3‐I	No (*p* > 0.05)	No (*p* > 0.05)
	p62 in Neuron	Yes (*p* < 0.0001) Greater in females in AD‐Veh and AD‐EVs group (*p* < 0.01–0.001)	No (*p* > 0.05)
	p62 in Microglia	No (*p* > 0.05)	No (*p* > 0.05)
Neurogenesis	BrdU	No (*p* > 0.05)	No (*p* > 0.05)
	BrdU‐NeuN	No (*p* > 0.05)	No (*p* > 0.05)
	Net Neurogenesis	No (*p* > 0.05)	No (*p* > 0.05)
	DCX	Yes (*p* < 0.01) Greater in males in AD‐EVs group (*p* < 0.05)	No (*p* > 0.05)
	BDNF	No (*p* > 0.05)	No (*p* > 0.05)
	p‐ERK1/2	Yes (*p* < 0.0001) Greater in females of all groups (*p* < 0.01–0.0001)	No (*p* > 0.05)
	p‐CREB	No (*p* > 0.05)	No (*p* > 0.05)

### 3.3 hiPSC‐NSC‐EVs Treatment Brought the Expression of Genes Encoding Proteins Involved in Maintaining Mitochondrial Respiratory Chain Closer to Naïve Control Levels in the Hippocampus

**FIGURE 2:**
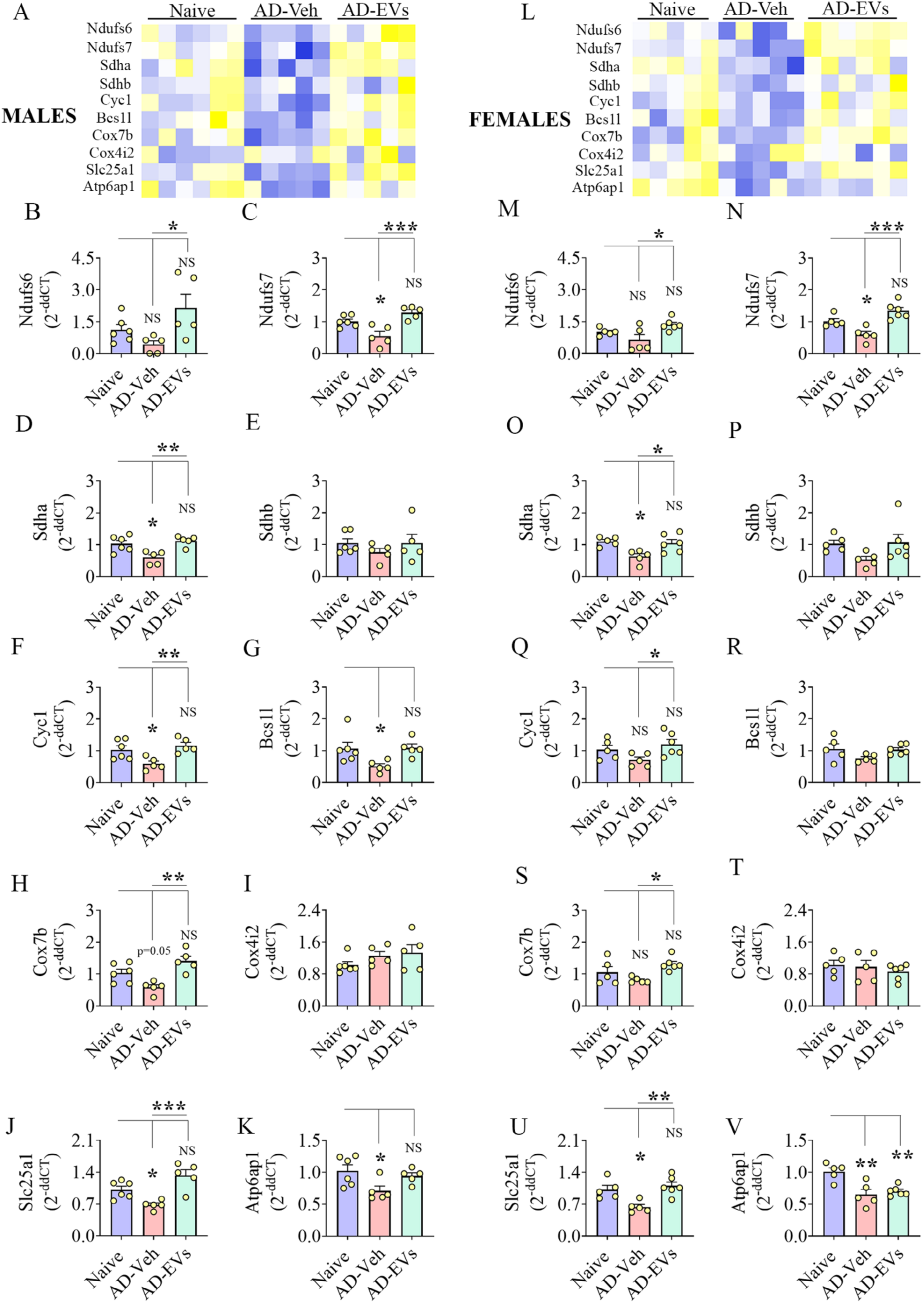
Intranasal administration of extracellular vesicles from human‐induced pluripotent stem cell‐derived neural stem cells (hiPSC‐NSC‐EVs) to 5xFAD mice restored the expression of genes involved in mitochondrial respiratory chain function to or near naïve control levels. Figures A (males) and L (females) illustrate heat maps of genes encoding proteins related to the mitochondrial respiratory chain in the hippocampus. Bar graphs (B–K) and (M–V) show the expression of genes linked to complex I: Ndufs6, Ndufs7 (B, C, M, N), complex II: Sdha, Sdhb (D, E, O, P), complex III: Cyc1, Bcs1l (F, G, Q, R), complex IV: Cox7b, Cox4i2 (H, I, S, T), and complex V: Slc25a1, Atp6ap1 (J, K, U, V) in male (B–K) and female (M–V) mice from different groups. *p < 0.05, **p < 0.01, and ***p < 0.001; NS, not significant.

We measured select genes encoding proteins linked to the function of complex I (Ndufs6, Ndufs7), complex II (Sdha, Sdhb), complex III (Cyc1, Bcs1l), complex IV (Cox7b, Cox4i2), and complex V (Slc25a1, Atp6ap1). In male and female AD‐Veh groups, the expression of most of these genes was downregulated compared to the respective naïve control groups. However, the expression of these genes in male and female AD‐EVs groups matched those in male and female naïve control groups (Figure 2A,L). The genes exhibiting significantly reduced expression in the male AD‐Veh group compared to the male naïve control group include Ndufs7, Sdha, Cyc1, Bcs1l, Cox7b, Slc25a1, and Atp6ap1 (*p* ≤ 0.05, Figure 2C,D,F–H,J,K). In the male AD‐EVs group, the expression of all these genes was similar to naïve control levels (*p* > 0.05, Figure 2C,D,F–H,J,K) with Ndufs7, Sdha, Cyc1, Cox7b, and Slc25a1 expression increasing above levels observed in the AD‐Veh group (*p* < 0.05–0.001, Figure 2C,D,F,H,J). In addition, Ndufs6 expression in the male AD‐EVs group was higher than in the male AD‐Veh group (*p* < 0.05, Figure 2B). The genes exhibiting significantly reduced expression in the female AD‐Veh group compared to the female naïve control group include Ndufs7, Sdha, Slc25a1, and Atp6ap1 (*p* < 0.05–0.01, Figure 2N,O,U,V). In the female AD‐EVs group, the expression levels of Ndufs7, Sdha, and Slc25a1 genes were found to be comparable to those in naïve control groups (*p* > 0.05) and increased compared to the female AD‐Veh group (*p* < 0.05–0.001, Figure 2N,O,U). In addition, the expression of Ndufs6, Cyc1, and Cox7b in the female AD‐EVs group was higher than in the female AD‐Veh group (*p* < 0.05, Figure 2M,Q,S). Thus, hiPSC‐NSC‐EVs treatment brought the expression of many genes encoding mitochondrial complex I‐V proteins involved in maintaining the mitochondrial electron transport chain in the hippocampus of male and female 5xFAD mice to naïve control levels. Moreover, a two‐way ANOVA analysis indicated that there were no sex‐related effects or interactions between sex and hiPSC‐NSC‐EVs treatment for most genes associated with the mitochondrial respiratory chain in 5xFAD mice. The only exception was Cox4i2, where male 5xFAD mice receiving EVs exhibited higher expression levels than female 5xFAD mice receiving EVs (Table 1).

**FIGURE 3:**
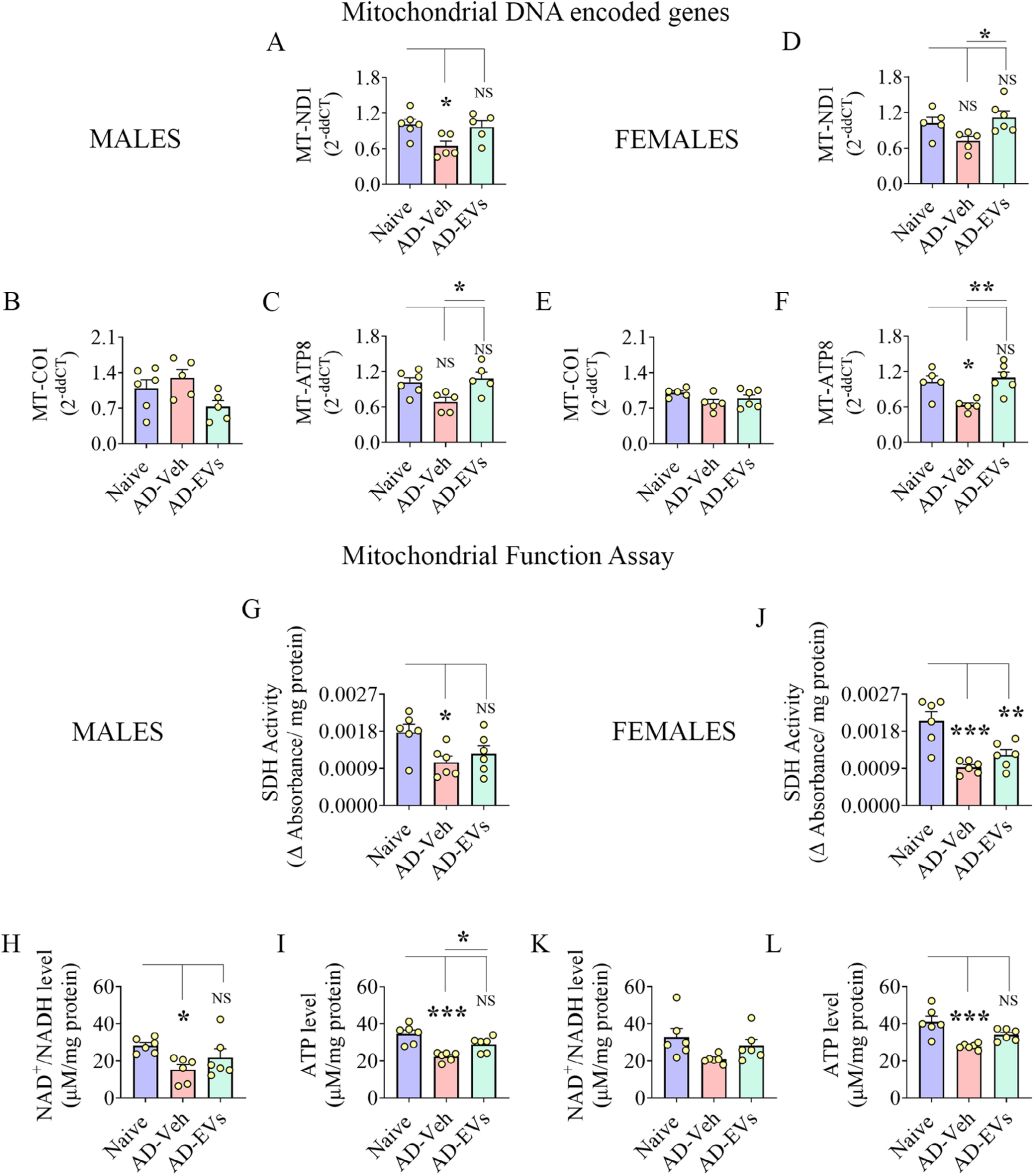
Intranasal administration of extracellular vesicles from human‐induced pluripotent stem cell‐derived neural stem cells (hiPSC‐NSC‐EVs) to 5xFAD mice enhanced the expression of genes linked to mitochondrial function in mitochondrial DNA and nuclear DNA. Bar charts A‐F compare the expression of mitochondrial DNA encoded genes, Mt‐Nd1 (A, D), Mt‐Co1 (B, E), and Mt‐Atp8 (C, F) in males (A–C) and females (D–F) across different groups. Bar charts (G–L) compare SDH activity (G, J), NAD+/NADH (H, K), and ATP (I, L) levels in males (G–I) and females (J–L) across different groups. *p < 0.05, **p < 0.01, and ***p < 0.001; NS, not significant.

We also measured mitochondrial DNA‐encoded genes such as Mt‐Nd1, Mt‐Co1, and Mt‐Atp8. In both males and females, Mt‐Nd1 expression was found to be reduced in AD‐Veh groups compared to naïve groups, but such differences were statistically significant only in males (*p* < 0.05, Figure 3A). In both male and female AD‐EVs groups, the level of Mt‐Nd1 expression was comparable to that seen in naïve groups (*p* > 0.05, Figure 3A,D). The expression of Mt‐Co1 did not differ across the three groups in both males and females (*p* > 0.05, Figure 3B,E). In contrast, Mt‐Atp8 expression was lower in the AD‐Veh groups than in the naïve groups, but such differences were statistically significant only in females (*p* < 0.05, Figure 3F). In both male and female AD‐EVs groups, the level of Mt‐Atp8 expression was comparable to that seen in naïve groups (*p* > 0.05), and significantly higher than in the AD‐Veh groups (*p* < 0.05–0.01, Figure 3C,F). Thus, the mitochondrial DNA‐encoded genes Mt‐Nd1 and Mt‐Atp8 were reduced in the AD‐Veh groups but were brought to naïve control levels in the AD‐EVs groups. The two‐way ANOVA analysis showed no sex‐specific effects on the efficacy of hiPSC‐NSC‐EVs in restoring the expression of mitochondrial DNA‐coded genes. However, there was an interaction between sex and treatment for Mt‐Co1, as its expression was relatively higher in male 5xFAD mice receiving hiPSC‐NSC‐EVs than in female 5xFAD mice receiving hiPSC‐NSC‐EVs (Table 1).

### 3.4 hiPSC‐NSC‐EVs Treatment Brought ATP Levels Closer to Naïve Control Levels in the Hippocampus

To evaluate potential improvements in mitochondrial function mediated by hiPSC‐NSC‐EVs, additional assays were conducted to measure SDH activity, NAD+/NADH ratios, and ATP levels in hippocampal tissue lysates from the experimental groups. In males, SDH activity, NAD+/NADH ratios, and ATP levels were significantly reduced in the AD‐Veh group compared to the naïve control group (*p* < 0.05–0.001, Figure 3G,I). In the AD‐EVs group, these markers matched those observed in the naïve control group (*p* > 0.05, Figure 3G–I), and overall ATP levels were also higher than in the AD‐Veh group (*p* < 0.05, Figure 3I). In female subjects, SDH activity and ATP levels were decreased in the AD‐Veh group relative to the naïve control group (*p* < 0.001; Figure 3J,L). NAD+/NADH ratios did not differ significantly among the naïve, AD‐Veh, and AD‐EVs groups in females (Figure 3K). In the AD‐EVs group, SDH activity remained lower (*p* < 0.01, Figure 3J), while ATP levels recovered to those of the naïve control group (*p* > 0.05, Figure 3L). The two‐way ANOVA analysis showed no sex‐specific effects on the efficacy of hiPSC‐NSC‐EVs in restoring the SDH and NAD/NADH activity. However, the levels of ATP were greater in female naïve and female 5xFAD mice receiving Veh (Table 1).

### 3.5 hiPSC‐NSC‐EVs Treatment Brought the Concentration of Proteins Involved in Mitochondrial Biogenesis, Fusion/Fission, and Mitophagy Closer to Naïve Control Levels in the Hippocampus

**FIGURE 4:**
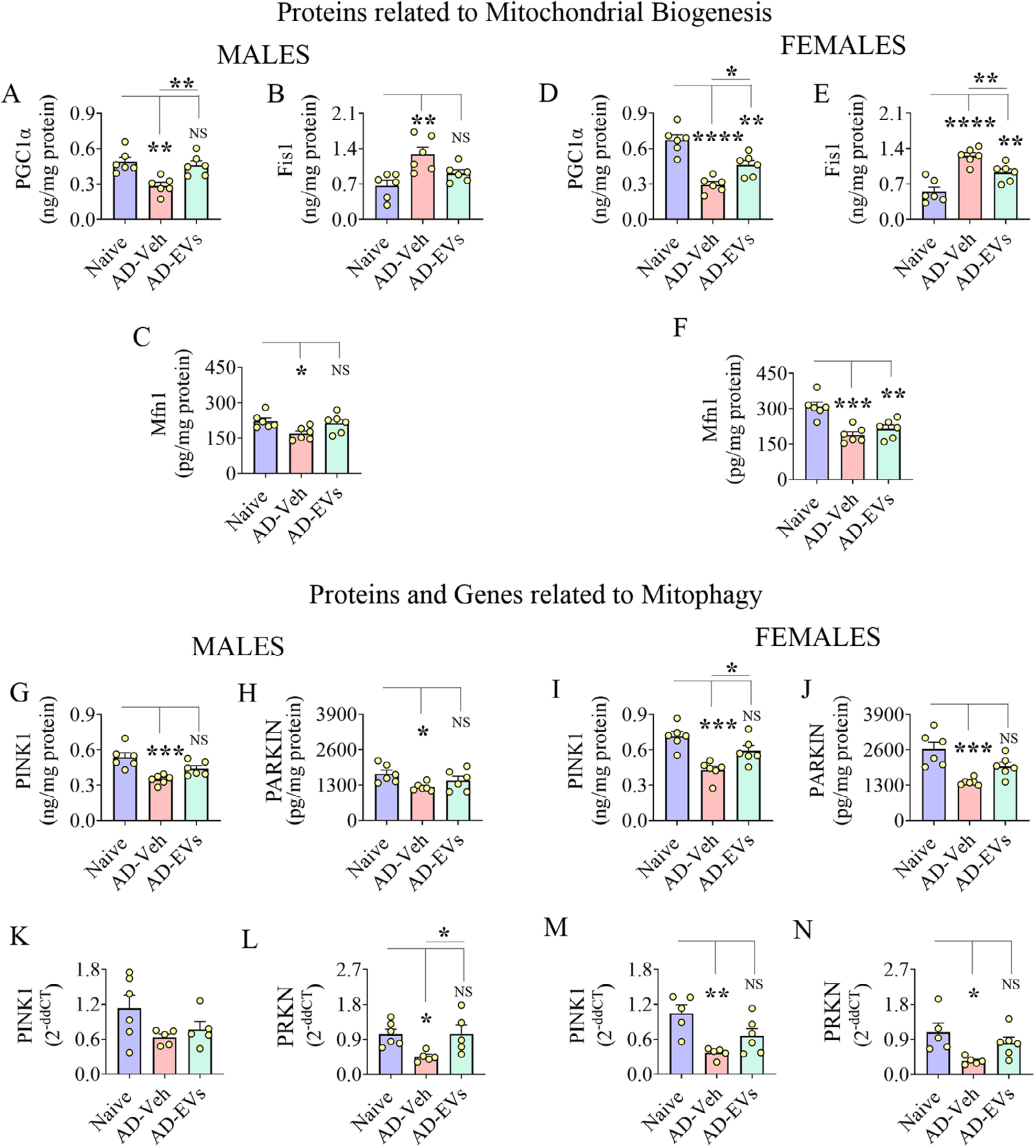
Intranasal administration of extracellular vesicles from human‐induced pluripotent stem cell‐derived neural stem cells (hiPSC‐NSC‐EVs) to 5xFAD mice restored protein levels involved in mitochondrial biogenesis and mitophagy to or near naive control levels. Bar charts (A–F) compare the concentrations of PGC‐1α (A, D), Fis1 (B, E), and Mfn1 (C, F) in male (A‐C) and female (D–F) mice between different groups. Figures G‐J compare mitophagy‐related proteins PINK1 (G, I) and PARKIN (H, J) in males (G, H) and females (I, J) across different groups. Bar charts K‐N compare the expression of mitophagy‐related genes PINK1 (K, M) and PARKN (L, N) in males (K, L) and females (M, N) across different groups *p < 0.05; **p < 0.01; and ***p < 0.001; ****p < 0.0001; NS, not significant.

The effects of hiPSC‐NSC‐EVs treatment on the concentrations of PGC‐1α, the primary regulator of mitochondrial biogenesis, as well as Mfn1 and Fis1, proteins involved in mitochondrial fusion and fission, and PINK1 and PARKIN, proteins involved in mitophagy, were evaluated. PGC‐1α concentrations were reduced in both male and female AD‐Veh groups compared to their respective naïve control groups (*p* < 0.01–0.0001, Figure 4A,D). In the male AD‐EVs group, PGC‐1α concentration matched that in the naïve control group (*p* > 0.05, Figure 4A). In both male and female AD‐EVs groups, PGC‐1α concentrations were significantly increased compared to the AD‐Veh groups (*p* < 0.05–0.01, Figure 4A,D). Fis1 concentrations were elevated in male and female AD‐Veh groups relative to their respective naïve control groups (*p* < 0.01–0.0001, Figure 4B,E). In the male AD‐EVs group, Fis1 concentration matched the male naïve control group (*p* > 0.05, Figure 4B), while in the female AD‐EVs group, Fis1 level was higher than the naïve control group but significantly decreased compared to the female AD‐Veh group (*p* < 0.01, Figure 4E). Contrastingly, the concentration of Mfn1 was decreased in male and female AD‐Veh groups compared to respective naïve control groups (*p* < 0.05–0.001, Figure 4C,F). In the male AD‐EVs group, Mfn1 concentration matched the naïve control group (*p* > 0.05, Figure 4C). In contrast, in the female AD‐EVs group, the Mfn1 level remained lower than that of the naive control group (*p* < 0.01, Figure 4F). WB analysis of DRP1 showed no differences across groups in males and females (*p* > 0.05; Figure S3A,B,D). The OPA1 levels were increased in the male AD‐EVs group compared to the male AD‐Veh group (*p* < 0.05, Figure S3C) but were not different across groups in females (*p* > 0.05, Figure S3E). The two‐way ANOVA analysis showed no sex‐specific effects on the efficacy of hiPSC‐NSC‐EVs in restoring the expression of DRP1 and OPA1. However, there was an interaction between sex and treatment for OPA1, as its expression was relatively higher in male 5xFAD mice receiving hiPSC‐NSC‐EVs than in female 5xFAD mice receiving hiPSC‐NSC‐EVs (Table 1).

The concentration of PINK1 was decreased in male and female AD‐Veh groups compared to respective naïve control groups (*p* < 0.001, Figure 4G,I). In both male and female AD‐EVs groups, PINK1 concentrations matched those of the naïve control groups (*p* > 0.05, Figure 4G,I); the female AD‐EVs group also displayed a higher concentration than the female AD‐Veh group (*p* < 0.05, Figure 4I). The concentration of PARKIN was also decreased in male and female AD‐Veh groups compared with their respective naïve control groups (*p* < 0.05–0.001; Figure 4H,J). In male and female AD‐EVs groups, PARKIN concentration matched that in respective naïve control groups (*p* > 0.05, Figure 4H,J). We also measured PINK1 and PRKN mRNA levels (Figure 4K–N). PINK1 gene expression did not vary across groups in males (Figure 4K); however, in females, compared to the naïve control group, the AD‐Veh group displayed reduced expression (*p* < 0.01), whereas its expression in the AD‐EVs group matched that in the naïve control group (*p* > 0.05, Figure 4M). PRKN gene expression was reduced in both male and female AD‐Veh groups compared to the naïve control group (*p* < 0.05) but matched levels in respective naïve control groups (*p* > 0.05, Figure 4L,N). PRKN expression in the male AD‐EVs group was also higher than that in the AD‐Veh group (*p* < 0.05, Figure 4L). Thus, hiPSC‐NSC‐EVs treatment in male and female 5xFAD mice brought back the concentrations of proteins involved in mitochondrial biogenesis, fission, fusion, and mitophagy to near naïve control levels in the hippocampus. A two‐way ANOVA revealed sex‐dependent differences in PGC‐1α, Mfn1, PINK1, and PARKIN. PGC‐1α and Mfn1 levels were significantly higher in naïve females than in naïve males. Additionally, PINK1 and PARKIN levels were higher in both naïve and AD‐EVs groups of female mice (Table 1).

### 3.6 hiPSC‐NSC‐EVs Treatment Brought p‐mTOR and p‐mTOR/Pan‐mTOR Ratio Closer to Naïve Control Levels in the Hippocampus

**FIGURE 5:**
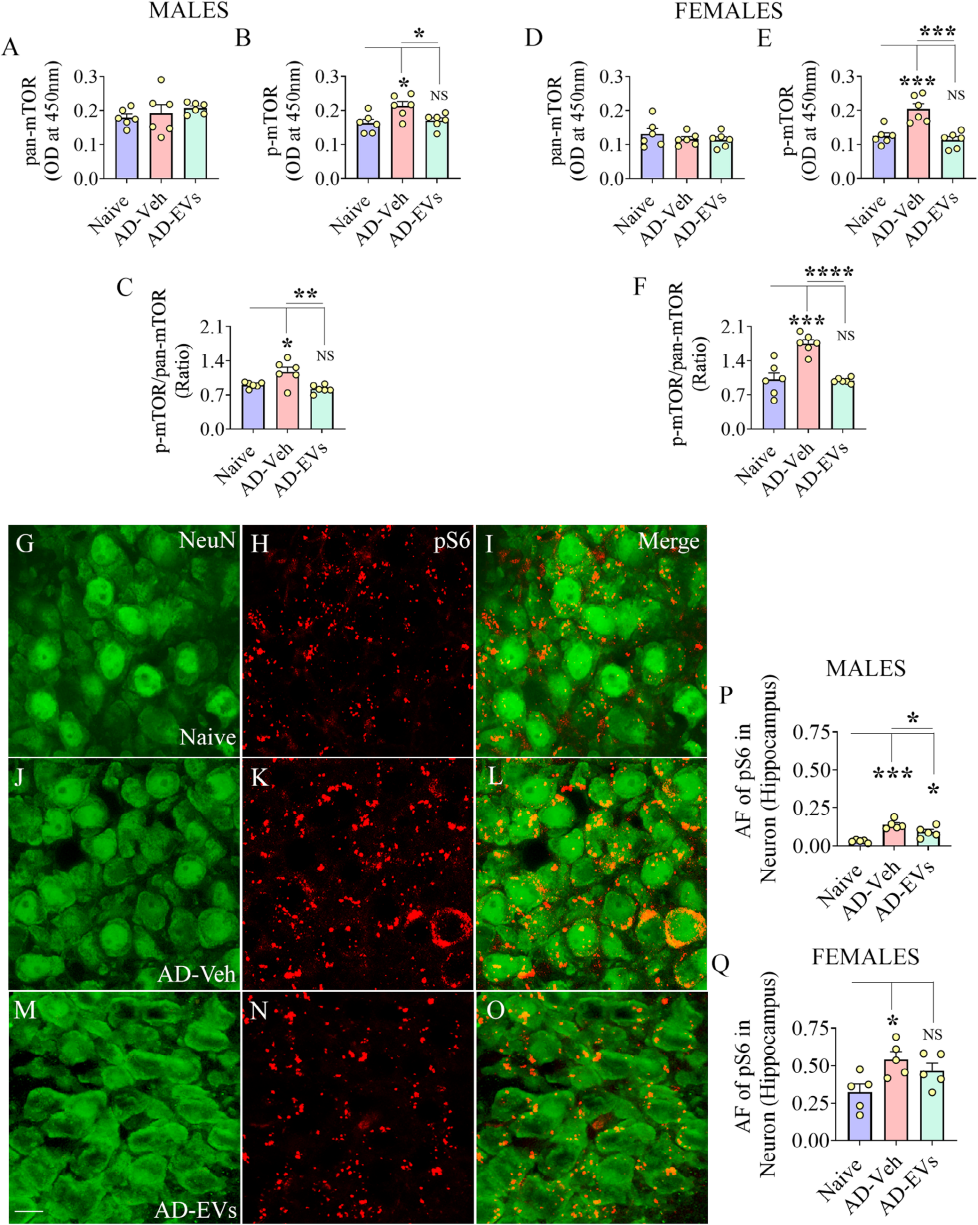
Intranasal administration of extracellular vesicles from human induced pluripotent stem cell‐derived neural stem cells (hiPSC‐NSC‐EVs) to 5xFAD mice reduced the levels of proteins involved in mTOR activation. Bar charts A‐F compare the concentrations of pan‐mTOR (A, D), p‐mTOR (B, E), and the ratio of p‐mTOR/pan‐mTOR (C, F) in the hippocampus of male (A–C) and female (D–F) mice across different groups. (G–O) illustrate examples of NeuN+ neurons (green) expressing pS6 (red) from naïve (G–I), AD‐Veh (J–L), and AD‐EVs (M–O) groups. Bar charts (P, Q) compare mean area fractions of pS6 in individual neurons from the CA3 sub‐region of the hippocampus across different groups. Scale bar, G–O = 10 μm. *p < 0.05, **p < 0.01, ***p < 0.001 and ****p < 0.0001; NS, not significant.

We measured the effects of hiPSC‐NSC‐EVs treatment on mTOR activation by quantifying pan‐mTOR, p‐mTOR, and the p‐mTOR/pan‐mTOR ratio in the hippocampus. While the concentration of pan‐mTOR did not differ between groups in both sexes (*p* > 0.05, Figure 5A,D), the level of p‐mTOR and the ratio of p‐mTOR/pan‐mTOR were significantly increased in both male and female AD‐Veh groups compared to the naïve control group (*p* < 0.05–0.001, Figure 5B,C,E,F). Contrastingly, in both male and female AD‐EVs groups, p‐mTOR concentration and the ratio of p‐mTOR/pan‐mTOR matched those in naïve control groups (*p* > 0.05) and were reduced compared to male and female AD‐Veh groups (*p* < 0.05–0.0001, Figure 5B,C,E,F). To confirm whether reduced p‐mTOR levels mediated by hiPSC‐NSC‐EVs treatment diminished the downstream mTOR signaling, we measured the extent of expression of pS6 in hippocampal CA3 pyramidal neurons and microglia, as a measure of mTORC1 activation (Arenas et al. 2020). Examples of hippocampal CA3 pyramidal neurons visualized with NeuN and pS6 dual immunofluorescence in male mice from the naïve, AD‐Veh, and AD‐EVs groups are shown (Figure 5G–O). Analysis of the area fraction of pS6 within individual CA3 pyramidal neurons suggested a significantly increased expression of pS6 in both male and female AD‐Veh groups (*p* < 0.05–0.001, Figure 5P,Q), implying the hyperactivation of mTOR signaling. In contrast, pS6 expression in the male AD‐EVs group was reduced compared to the male AD‐Veh group (*p* < 0.05, Figure 5P), whereas pS6 expression in the female AD‐EVs group matched that in the female naïve control group (*p* > 0.05, Figure 5Q). Additionally, we measured the percentage of microglia expressing pS6. Examples of microglia expressing pS6 visualized with Iba1 and pS6 dual immunofluorescence in male mice from the naïve, AD‐Veh, and AD‐EVs groups are shown in Figure S4A–I. In both males and females, the percentage of microglia expressing pS6 was significantly increased in the AD‐Veh groups compared to the respective naïve groups (*p* < 0.001–0.0001; Figure S4J,K). However, in males, the percentage of microglia expressing pS6 was found to be reduced with EV treatment compared to the AD‐Veh group (*p* < 0.001), and it was similar to that of the naïve group in females (*p* > 0.05, Figure S4J,K). Thus, hiPSC‐NSC‐EVs treatment in male and female 5xFAD mice alleviated the hyperactivation of mTOR signaling within neurons and microglia in the hippocampus. Two‐way ANOVA analysis showed sex‐dependent differences for pan‐mTOR, p‐mTOR, p‐mTOR/pan‐mTOR ratio, and pS6. Males displayed higher concentrations of p‐mTOR in the naïve and AD‐EVs group and pan‐mTOR in all three groups. In addition, the ratio was significantly higher in the female AD‐Veh group. There was an interaction between sex and EVs treatment, with greater effects in females. The pS6 level showed sex‐specific differences, with females exhibiting greater expression in all groups (Table 1).

### 3.7 hiPSC‐NSC‐EVs Treatment Brought the Concentration of Several Proteins Linked to Autophagy Closer to Naïve Control Levels in the Hippocampus

**FIGURE 6:**
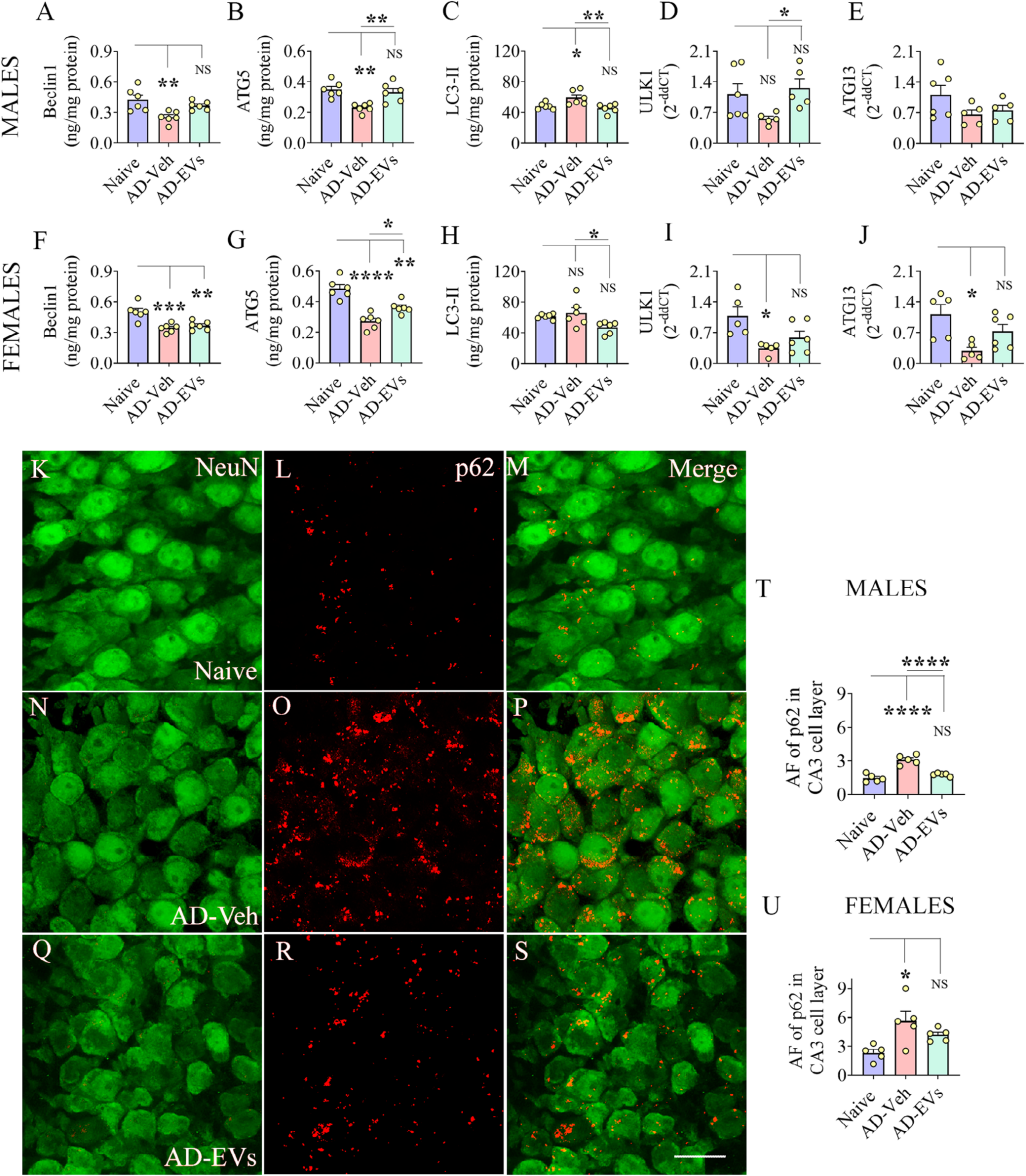
Intranasal administration of extracellular vesicles derived from human induced pluripotent stem cell‐derived neural stem cells (hiPSC‐NSC‐EVs) to 5xFAD mice restored autophagy‐related proteins in the hippocampus to or near naive control levels. Bar charts (A–J) compare the concentrations of beclin‐1 (A, F), ATG‐5 (B, G), and LC3‐II (C, H) and gene expression levels of ULK1 (D, I) and ATG13 (E, J) in the hippocampus of males (A–E) and females (F–J) across different groups. The images in K‐S illustrate examples of NeuN+ (green) neurons expressing p62 (red) in CA3 pyramidal neurons from naïve (K–M), AD‐Veh (N–P), and AD‐EVs (Q–S) groups. The bar charts T‐U compare area fractions of p62+ structures in the CA3 pyramidal cell layer of the hippocampus. Scale bar, K–S = 10 μm. *p < 0.05, **p < 0.01, ***p < 0.001 and ****p < 0.0001; NS, not significant. Illustration of a negative control brain tissue section processed while performing NeuN and p62 dual immunofluorescence, with the omission of p62 antibody, is available in Figure S8.

We measured the consequences of the hiPSC‐NSC‐EVs treatment‐mediated downregulation of mTOR signaling on the concentrations of several proteins contributing to autophagy, including beclin‐1, ATG‐5, and LC3‐II. The concentrations of beclin‐1 and ATG‐5 were decreased in male and female AD‐Veh groups compared to respective naïve control groups (*p* < 0.01–0.0001, Figure 6A,B,F,G). In the male AD‐EVs group, the concentrations of beclin‐1 and ATG‐5 matched those in the naïve control group (*p* > 0.05, Figure 6A,B), with ATG‐5 showing significantly increased concentration than the AD‐Veh group (*p* < 0.01, Figure 6B). In the female AD‐EVs group, compared to the AD‐Veh group, the concentration of beclin‐1 did not change (*p* > 0.05, Figure 6F), but the concentration of ATG‐5 increased (*p* < 0.05, Figure 6G). On the other hand, compared to naïve control groups, the concentrations of LC3‐II increased in the male AD‐Veh group (*p* < 0.05, Figure 6C) but not in the female AD‐Veh group (*p* > 0.05, Figure 6H). Nonetheless, the concentrations of LC3‐II decreased in both male and female AD‐EVs groups compared to respective AD‐Veh groups (*p* < 0.05–0.01, Figure 6C,H).

We also measured ULK1 and ATG13 gene expression using qPCR (Figure 6D,E,I,J). ULK1 expression, compared with naive control groups, was reduced in both male and female AD‐Veh groups (Figure 6D,I), with a significant reduction in the female AD‐Veh group (*p* < 0.05). In the AD‐EVs groups, ULK1 expression matched that in the naive control groups (*p* > 0.05, Figure 6D,I), with the male AD‐EVs group displaying higher expression than the AD‐Veh group (*p* < 0.05, Figure 6D). ATG13 expression did not differ across groups in males (*p* > 0.05, Figure 6E); however, in females, compared to the naive control group, ATG13 expression decreased in the AD‐Veh group (*p* < 0.05, Figure 6J) but was similar in the AD‐EVs group (*p* > 0.05, Figure 6J). Western blot analysis for LC3‐I and LC3‐II proteins and their ratio did not show differences in males across groups (Figure S5A–D). In females, compared to the naive control group, the AD‐EVs group displayed reduced LC3‐1 (*p* < 0.05, Figure S5E), no changes in LC3‐II (*p* > 0.05, Figure S5F), but a higher LC3‐II/LC3‐I ratio (*p* < 0.05, Figure S5G). The LC3‐II/LC3‐I ratio was also higher in the AD‐EVs group compared to the AD‐Veh group (*p* < 0.05, Figure S5G). Overall, hiPSC‐NSC‐EVs treatment brought the levels of beclin‐1, ATG‐5, and LC3‐II proteins and ULK1 gene expression closer to naive control levels in male 5xFAD mice. In female 5xFAD mice, hiPSC‐NSC‐EVs treatment brought LC3‐II protein levels and ULK1 and ATG13 gene expression closer to naïve control levels, and enhanced ATG5 expression compared to Veh‐treated counterparts. Two‐way ANOVA showed higher beclin1 in females within the AD‐Veh groups, and higher ATG5 and LC3‐II in females within the naïve groups. There was also an interaction between sex and EVs treatment for ATG‐5, with males showing greater effects.

To further confirm the autophagy‐enhancing effects of hiPSC‐NSC‐EVs in the hippocampus of male and female 5xFAD mice, we measured the amount of p62 in CA3 pyramidal neurons. The protein p62, a cargo adapter that binds to ubiquitinated proteins to facilitate their delivery to autophagosomes for degradation, gets degraded along with its cargo. Hence, reduced p62 levels indicate active autophagy, whereas increased p62 accumulation will indicate reduced autophagy (Liu et al. 2016). Examples of hippocampal CA3 pyramidal neurons visualized with NeuN and p62 dual immunofluorescence from male mice belonging to naïve, AD‐Veh, and AD‐EVs groups are illustrated (Figure 6K–S). Analysis of the area fraction of p62 immunofluorescent particles in the CA3 pyramidal cell layer suggested a significantly increased expression of p62 in both male and female AD‐Veh groups (*p* < 0.05–0.0001, Figure 6T,U), implying reduced autophagy. In contrast, p62 expression in male and female AD‐EVs groups was brought to levels in respective naïve control groups (*p* > 0.05, Figure 6T,U), with the male AD‐EVs group showing substantially less p62 than the male AD‐Veh group (*p* < 0.0001, Figure 6T). Thus, evaluation of the extent of p62+ structures suggested that hiPSC‐NSC‐EVs treatment in male and female 5xFAD mice improved autophagy in hippocampal neurons. Two‐way ANOVA revealed higher levels of p62 in females with both AD‐Veh and AD‐EVs groups.

### 3.8 hiPSC‐NSC‐EVs Treatment Prevented the Decline in Hippocampal Neurogenesis

**FIGURE 7:**
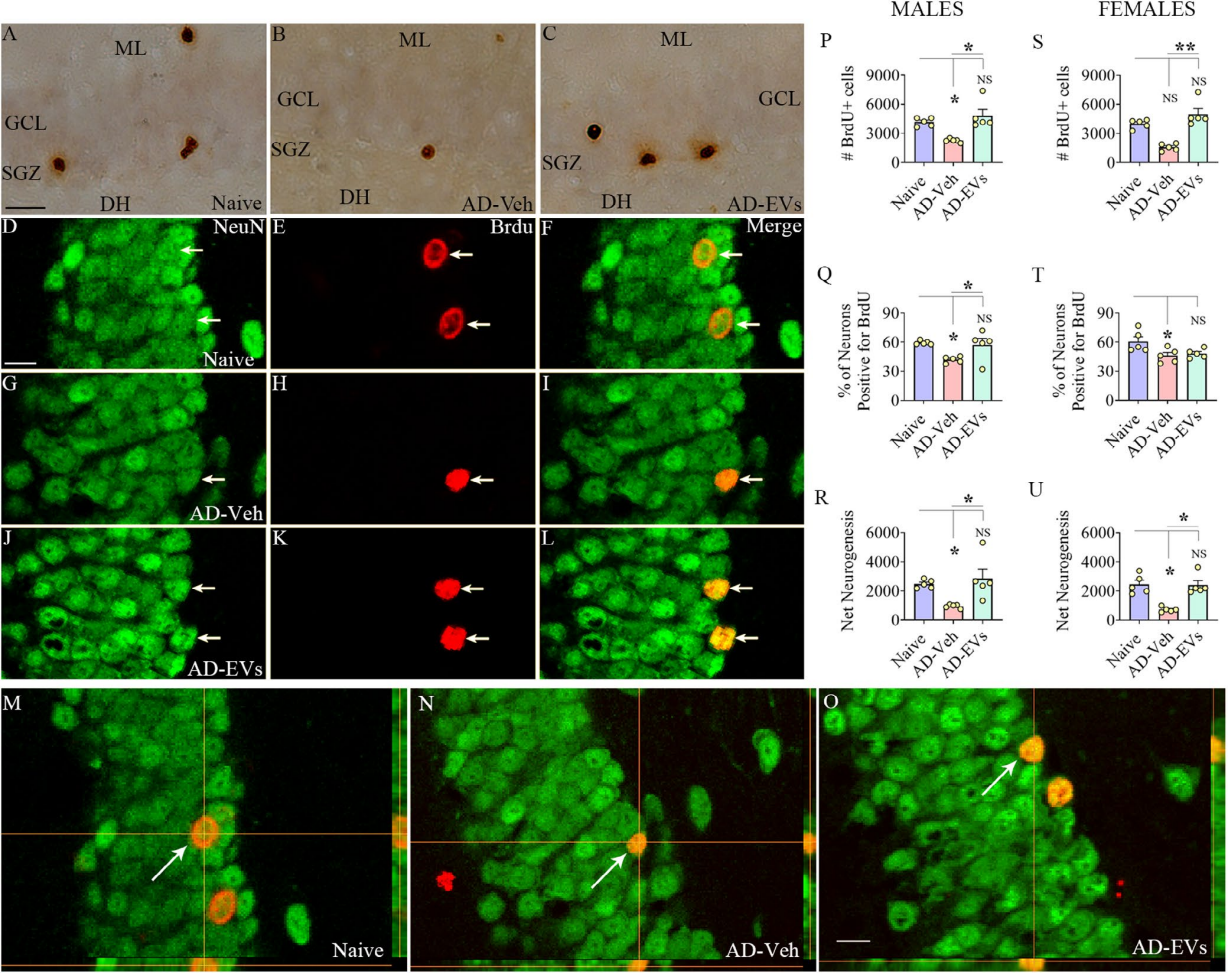
Intranasal human induced pluripotent stem cell‐derived neural stem cells (hiPSC‐NSC‐EVs) treatment in 5XFAD mice alleviated hippocampal neurogenesis decline at 4 months of age. The images in (A–C) illustrate 5′‐bromodeoxyuridine‐positive (BrdU+) cells in the subgranular zone‐ granular cell layer (SGZ‐ GCL) from naive (A), AD‐Veh (B), and AD‐EVs (C) groups. The images in (D–L) illustrate examples of BrdU+ newly born cells (red) that differentiated into NeuN+ mature neurons (green) from naive (D–F), AD‐Veh (G–I), and AD‐EVs (J–L) groups. The images in M‐O show orthogonal views of selected neurons (indicated by arrows) from (F, I, and L). Bar charts compare BrdU+ cell counts in males (P) and females (S), percentage of BrdU+ neurons expressing NeuN in males (Q) and females (T), and net hippocampal neurogenesis in males (R) and females (U). Scale bars, A–C = 25 μm; D–L, M–O = 10 μm. *p < 0.05, and **p < 0.01; NS, not significant.

We measured the effects of hiPSC‐NSC‐EVs treatment on net hippocampal neurogenesis by BrdU labeling for 7 days a week after administering the second dose of EVs (~at 3.5 months of age). The surviving newly generated cells (BrdU+ cells) were visualized ~1.5 months later using BrdU immunostaining (Figure 7A–C), and their differentiation into neurons was determined via BrdU‐NeuN dual immunofluorescence (Figure 7D–L). Orthogonal views of newly added neurons showing both NeuN and BrdU are illustrated in Figure 7M–O. Stereological quantification of BrdU+ cells in the SGZ‐GCL of the hippocampus revealed fewer newly added cells in the male AD‐Veh group compared to the respective naïve control group (*p* < 0.05, Figure 7P). A similar trend was observed in the female AD‐Veh group (*p* > 0.05, Figure 7S). Notably, in male and female AD‐EVs groups, the numbers of newly added cells were comparable to those in respective naïve control groups (*p* > 0.05) and higher than the numbers in AD‐Veh groups (*p* < 0.05–0.01, Figure 7P,S). Compared to naïve control groups, percentages of newly added cells differentiating into neurons were lower in male and female AD‐Veh groups (*p* < 0.05, Figure 7Q,T) but similar in male and female AD‐EVs groups (*p* > 0.05, Figure 7Q,T), with the male AD‐EVs group showing a higher level of neuronal differentiation of newly added cells than the male AD‐Veh group (*p* < 0.05, Figure 7Q). Next, we measured net hippocampal neurogenesis using data such as the numbers of BrdU+ cells and the percentages of neuronal differentiation of BrdU+ cells. Such analysis revealed decreased net neurogenesis in male and female AD‐Veh groups compared to respective naïve control groups (*p* < 0.05, Figure 7R,U). However, male and female AD‐EVs groups displayed similar net hippocampal neurogenesis as respective naïve control groups (*p* > 0.05, Figure 7R,U) and higher levels of neurogenesis than male and female AD‐Veh groups (*p* < 0.05, Figure 7R,U). Thus, hiPSC‐NSC‐EVs treatment in male and female 5xFAD mice prevented the decline in net hippocampal neurogenesis. Two‐way ANOVA analysis revealed no sex‐dependent differences or interactions between sex and EVs treatment.

**FIGURE 8:**
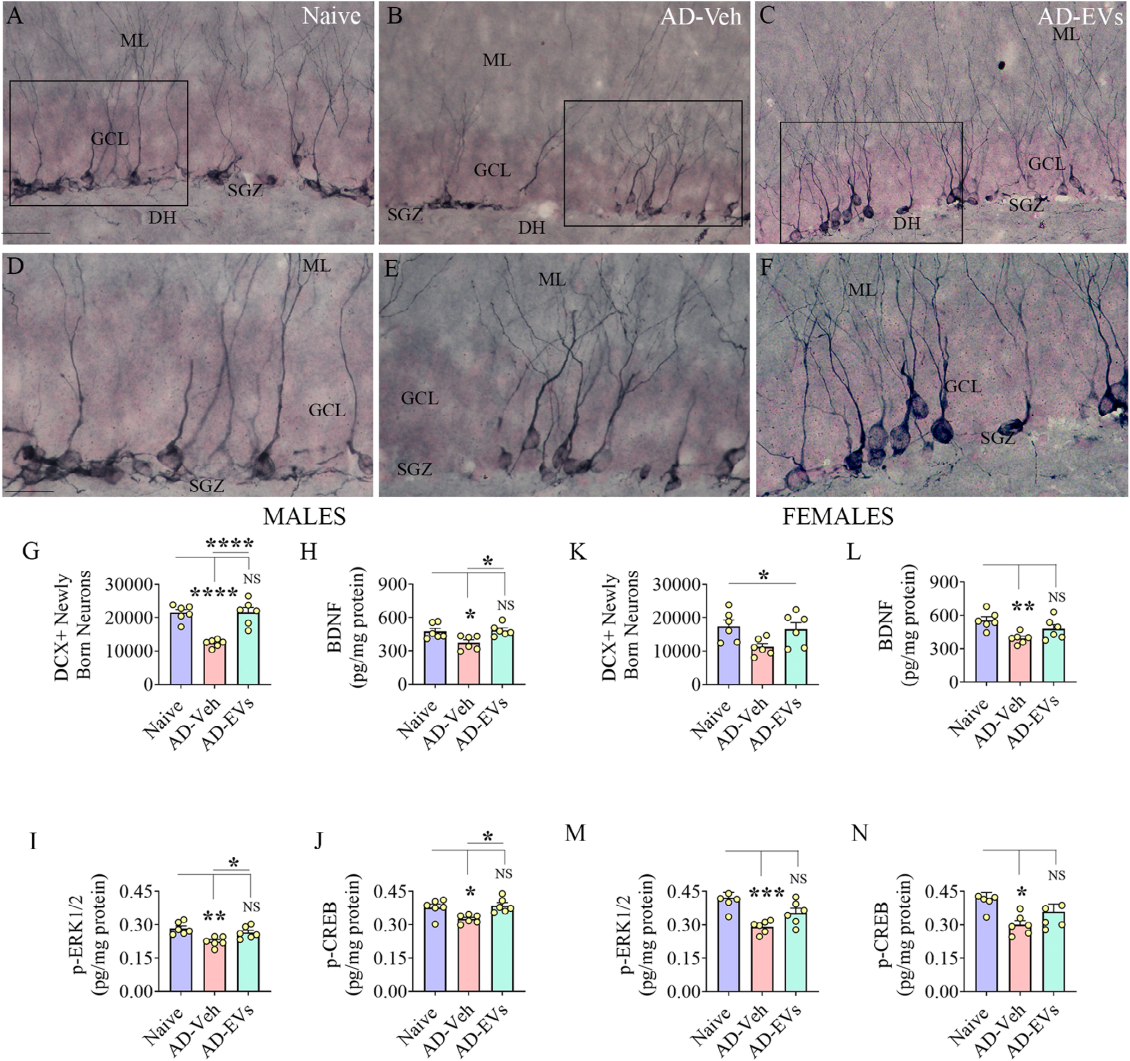
Intranasal human induced pluripotent stem cell‐derived neural stem cells (hiPSC‐NSC‐EVs) treatment to 5XFAD mice maintained higher levels of hippocampal neurogenesis. The images in (A–F) illustrate doublecortin‐positive (DCX+) newly born cells from naive (A, D), AD‐Veh (B, E), and AD‐EVs (C, F) groups in the subgranular zone‐granule cell layer (SGZ‐GCL). The images in (D–F) illustrate magnified views of regions from figures A, B, and C. Bar charts in G (males) and K (females) compare the numbers of DCX+ neurons in the SGZ‐GCL between different groups. The bar charts in H–J and L–N compare the concentrations of BDNF (H, L), p‐ERK (I, M), and p‐CREB (J, N) in male (H–J) and female (L–N) mice across different groups. Scale bar, A–C = 50 μm, D–F = 25 μm. *p < 0.05, **p < 0.01, ***p < 0.001 and ****p < 0.0001; NS, not significant.

To examine whether hiPSC‐NSC‐EVs treatment at 3 months of age in male and female 5xFAD mice would maintain similar levels of new neuron production as naïve control mice in the SGZ‐GCL at 5 months of age, we visualized newly born neurons via DCX immunostaining. DCX+ immature neurons in the SGZ‐GCL of the hippocampus in rodents represent new neurons born during the 2–3 weeks prior to euthanasia and hence provide a measure of the status of new neuron production (Rao and Shetty 2004; Brown et al. 2003). Examples of DCX+ newly born neurons in the SGZ‐GCL from male mice belonging to naïve, AD‐Veh, and AD‐EVs groups are illustrated (Figure 8A–F). Stereological quantification of DCX+ neurons in the SGZ‐GCL revealed reduced production of new neurons in the male AD‐Veh group compared to the male naïve control group (*p* < 0.0001, Figure 8G). However, the male AD‐EVs group maintained similar production of new neurons as the male naïve control group (*p* > 0.05, Figure 8G). Moreover, hiPSC‐NSC‐EVs treatment increased the production of new neurons in males compared to the AD‐Veh group (*p* < 0.0001, Figure 8G). Compared to the female naïve control group, the production of new neurons in the female AD‐Veh and AD‐EVs groups showed a similar trend to that observed in their male counterparts. However, post hoc tests did not reveal differences between groups (*p* > 0.05, Figure 8K). Thus, hiPSC‐NSC‐EVs treatment in male and female 5xFAD mice maintained similar levels of new neuron production as naïve control mice at 5 months of age. Two‐way ANOVA revealed higher numbers of DCX+ neurons in males within the AD‐EVs group.

### 3.9 hiPSC‐NSC‐EVs Treatment Alleviated BDNF‐ERK‐CREB Signaling Decline in the Hippocampus

Since BDNF concentration wanes significantly in the hippocampus of AD patients and in 5xFAD mice (Choi et al. 2018; Gao et al. 2022), likely due to Aβ‐induced inhibition of CREB phosphorylation (Vitolo et al. 2002), we investigated the effects of hiPSC‐NSC‐EVs treatment on the maintenance of BDNF levels and BDNF‐ERK‐CREB signaling in the hippocampus. The concentrations of BDNF, p‐ERK, and p‐CREB were decreased in male and female AD‐Veh groups compared to respective naïve control groups (*p* < 0.05–0.001, Figure 8H–J,L–N). Notably, in both male and female AD‐EVs groups, the concentrations of BDNF, p‐ERK, and p‐CREB matched those in respective naïve control groups (*p* > 0.05, Figure 8H–J,L–N). In addition, their concentrations were significantly higher in the male AD‐EVs group than in the AD‐Veh group (*p* < 0.05, Figure 8H–J). Thus, hiPSC‐NSC‐EVs treatment in male and female 5xFAD mice maintained similar BDNF concentrations and BDNF‐ERK‐CREB signaling as respective naïve control mice. Two‐way ANOVA revealed no sex‐specific differences, except for p‐ERK1/2, which was higher in females across all groups.

## 4 Discussion

The results of this study offer novel insights into the effectiveness of hiPSC‐NSC‐EVs in reversing or mitigating pathological changes associated with AD when administered intranasally during the early disease stage. First, hiPSC‐NSC‐EVs treatment reduced oxidative stress by enhancing antioxidant activity. Second, the intervention reversed or attenuated detrimental changes in the expression of proteins and genes that maintain mitochondrial health, including genes encoding mitochondrial complex proteins and proteins involved in mitochondrial biogenesis, fission, fusion, and mitophagy. Third, the treatment brought the concentrations of proteins associated with mTOR signaling overactivation and reduced autophagy to levels comparable to those of naïve controls. Fourth, the intervention maintained higher levels of hippocampal neurogenesis and upregulated BDNF.

### 4.1 hiPSC‐NSC‐EVs Treatment to 5xFAD Mice Reduced Oxidative Stress and Improved Mitochondrial Health in the Hippocampus

We found enhanced oxidative stress in the hippocampus of 5xFAD mice, consistent with the previous finding (Kim et al. 2019). Increased oxidative stress was particularly prominent in male 5xFAD mice from significantly elevated levels of MDA, a product of lipid peroxidation serving as a marker of oxidative damage (Cordiano et al. 2023), and PCs, a measure of protein carbonylation mediated by ROS (Dalle‐Donne et al. 2003). Notably, hiPSC‐NSC‐EVs treatment induced reductions in MDA and PCs in male mice, along with normalization in the concentrations of NRF‐2, a transcription factor involved in cellular defense against oxidative stress (Buendia et al. 2016), and catalase, an antioxidant (Nandi et al. 2019). While female 5xFAD mice showed a similar trend, the elevations in concentrations of MDA and PCs were not statistically significant compared to naïve mice. Nonetheless, female 5xFAD mice receiving hiPSC‐NSC‐EVs treatment displayed reduced concentrations of MDA and PCs, with an increased concentration of NRF‐2, compared to their counterparts receiving the Veh. Furthermore, we observed reduced expression of multiple genes encoding mitochondrial complex proteins involved in maintaining the mitochondrial electron transport chain in 5xFAD mice, which is consistent with the concept that increased oxidative stress can damage mitochondria, resulting in reduced energy production and increased ROS generation, further exacerbating oxidative stress (Ionescu‐Tucker and Cotman 2021). Treatment with hiPSC‐NSC‐EVs brought the expression of several genes encoding mitochondrial complex I‐V proteins in the hippocampus of both male and female 5xFAD mice to or near naïve control levels.

Next, we confirmed that, in the hippocampus of both male and female 5xFAD mice, levels of PGC‐1α and Mfn1 (regulators of mitochondrial biogenesis and fusion) were reduced, alongside decreased Fis1 (a mitochondrial fission protein) and PINK1 and PARKIN, proteins that promote mitophagy. Treatment with hiPSC‐NSC‐EVs normalized all protein levels in male 5xFAD mice. In female 5xFAD mice, hiPSC‐NSC‐EVs treatment increased PGC‐1α and Mfn1 and decreased Fis1 compared to Veh‐treated counterparts. This treatment also brought PINK1 and PARKIN levels to those in the naïve controls. Thus, despite minor sex‐dependent effects, the findings suggest that hiPSC‐NSC‐EVs treatment can bring back the levels of multiple proteins that maintain mitochondrial health in both male and female 5xFAD mice to or near naïve control levels, which is significant since cognitive decline in AD is associated with mitochondrial impairments characterized by reduced biogenesis and mitophagy (McGill Percy et al. 2025; D'Alessandro et al. 2025).

The proficiency of hiPSC‐NSC‐EVs to reduce oxidative stress and improve mitochondrial function in 5xFAD mice observed in this study is consistent with our recent cell culture study demonstrating that hiPSC‐NSC‐EVs treatment can suppress oxidative stress in mature human neurons challenged with Aβ‐42 oligomers by reducing total ROS production and mitochondria‐generated superoxide (Rao et al. 2025). The antioxidant and mitochondria‐protecting effects of hiPSC‐NSC‐EVs may be linked to hemopexin, which is one of the highly enriched proteins in these EVs, based on our previous proteomic studies (Upadhya et al. 2020). As hemopexin forms a stable complex with heme, it can prevent heme‐mediated production of ROS and thereby thwart oxidative damage to lipids and proteins (Li et al. 2009; Leclerc et al. 2018; Stalder et al. 2026). Such an effect is consistent with the reduced concentration of MDA and PCs observed in the hippocampus of both male and female 5xFAD mice receiving hiPSC‐NSC‐EVs in this study. However, it remains to be determined in future studies whether the levels of hemopexin or other heme‐handling proteins in 5xFAD brain could be altered by hiPSC‐NSC‐EVs treatment. Additional factors influencing reduced oxidative stress and improved mitochondrial health in 5xFAD mice receiving hiPSC‐NSC‐EVs treatment likely include reduced microglia‐mediated chronic neuroinflammation as reported in our recent study (Madhu et al. 2024). Such a likelihood is supported by the observations that reduced neuroinflammation decreases oxidative stress, as these two pathologies are tightly linked in a self‐perpetuating cycle where each process can induce and exacerbate the other (Fabisiak and Patel 2022; Dmytriv et al. 2024). Furthermore, alleviation of chronic neuroinflammation involved modulating activated microglia to a less inflammatory state (Madhu et al. 2024), which can make the brain's microenvironment less conducive to generating ROS, resulting in homeostasis between ROS and antioxidant defenses (Dmytriv et al. 2024).

### 4.2 hiPSC‐NSC‐EVs Treatment in 5xFAD Mice Reduced mTOR Overactivation and Increased Autophagy in the Hippocampus

The hippocampus of both male and female 5xFAD mice treated with Veh showed mTOR hyperactivation and reduced autophagy. The overactivation of mTOR could be confirmed from increases in p‐mTOR concentration, p‐mTOR/pan‐mTOR ratio, and pS6 expression in hippocampal neurons, with no changes in total mTOR concentration. In line with these findings, autophagy was impaired in both male and female 5xFAD mice receiving Veh treatment, as mTOR overactivation impairs the ULK1 protein complex, which plays a critical role in initiating and regulating autophagy (Cai et al. 2015). Indeed, compared to the naïve control group, ULK1 gene expression was reduced in the AD‐Veh groups but not in the AD‐EVs groups. Autophagy impairment in 5xFAD mice receiving Veh treatment was apparent from several measures. These mice displayed decreased levels of beclin 1, a protein involved in autophagy initiation and reduced in AD (Pickford et al. 2008), and ATG5, a protein essential for the formation and extension of the autophagosome membrane and reduced in AD (Ye et al. 2018; Longobardi et al. 2024). In addition, the expression of the ATG13 gene, encoding an adaptor protein that regulates ULK1 and acts as a crucial switch for initiating autophagy (Hama et al. 2025), was also reduced in female 5xFAD mice receiving Veh. However, the expression of LC3‐II, a protein involved in the biogenesis of autophagosomes, which is upregulated in AD to clear Aβ and p‐tau when autophagic flux is impaired (Lee and Lee 2016; Long et al. 2020), was inconclusive. While ELISA measurements revealed reduced LC3‐II concentrations in both male and female 5xFAD mice receiving EVs compared to those receiving Veh, WB analysis of the LC3‐I to LC3‐II conversion revealed no differences among males across groups, and in females, the AD‐EVs group showed a higher ratio than the naïve and AD‐Veh groups. Also, there was a discrepancy in LC3‐II levels between ELISA and WB measurements in both males and females. Nonetheless, the indication of improved autophagy in the AD‐EVs groups, as gleaned from measurements of beclin‐1 and ATG5, could be corroborated by increased expression of p62 in the AD‐Veh group compared to the naïve control group, and by comparable p62 expression in the AD‐Veh group to that in the naïve control group. This is because decreased levels of p62 in the AD‐EVs group imply better autophagy, as p62 is degraded as autophagosomes are delivered to lysosomes for degradation (Pankiv et al. 2007). Overall, the interpretation of reduced autophagy in the AD‐Veh groups and improved autophagy in the AD‐EVs groups in this study is based on measurements of markers, Beclin‐1, ATG5, ULK1, ATG13, and p62. However, the extent of autophagy enhancement mediated by hiPSC‐NSC‐EVs needs to be validated in future studies by measuring autophagic flux.

The above findings are consistent with studies on 5xFAD mouse brain reporting mTOR hyperactivation (Barbour et al. 2024), decreased beclin‐1 levels (Companys‐Alemany et al. 2022), and increased p62 expression (Raha et al. 2021). Notably, in both male and female 5xFAD mice, hiPSC‐NSC‐EVs treatment reduced p‐mTOR levels and the p‐mTOR/pan‐mTOR ratio, and decreased pS6 expression in hippocampal neurons and microglia, suggesting alleviation of mTOR overactivation. This modulation of mTOR signaling was also associated with increased levels of beclin 1 and ATG5 and reduced levels of p62, suggesting improved autophagy in the hippocampus as a whole. However, it remains to be determined whether improved autophagy is observed in all neural cell types, including glia, as p62 expression was measured only in neurons in this study.

The above findings raise an important question as to how hiPSC‐NSC‐EVs modulate mTOR signaling and autophagy. A careful examination of miRNA composition of hiPSC‐NSC‐EVs from our previous study (Upadhya et al. 2020) revealed the presence of multiple miRNAs, such as miR‐122, miR‐218‐2, miR‐99b, and miR‐16, capable of reducing mTOR activation by targeting the various steps of mTOR signaling. For example, miR‐122 can reduce phosphoinositide 3‐kinase (PI3K) concentration directly by inhibiting its subunit phosphatidylinositol‐4,5‐bisphosphate 3‐kinase catalytic subunit gamma or indirectly by inhibiting insulin‐like growth factor‐1 receptor (IGFR) and vascular endothelial growth factor C (Zhang et al. 2017; Wang et al. 2012). Similarly, miR‐218 can reduce the concentrations of PI3K directly by inhibiting the mTOR complex component Rictor and PI3K subunits PIK3C2A and PIK3R1 and can reduce AKT concentration indirectly by inhibiting the synthesis of mTORC2 (Zhang et al. 2017; Uesugi et al. 2011). Furthermore, miR‐99 family (a and b) can directly reduce the concentration of mTORC1 via direct targeting of its 3′‐UTR in a post‐transcriptional manner and indirectly by reducing the concentration of PI3K by inhibiting IGFR (Zhang et al. 2017; Cao et al. 2017). Additionally, miR‐16 can inhibit mTOR activation by directly targeting the 3′UTR of Rictor, a core component of mTORC2 complex (Huang et al. 2015). Thus, it is likely that multiple miRNAs in the cargo of hiPSC‐NSC‐EVs played a role in reducing mTOR hyperactivation in 5xFAD mice, which, in turn, led to improved autophagy. These results also suggest that delivering these miRNAs to the AD brain is likely therapeutic. Additionally, reduced microglia‐mediated chronic neuroinflammation as reported in our recent study (Madhu et al. 2024) may have also played a role in reducing mTOR activation, as previous studies imply that suppression of chronic neuroinflammation can reduce mTOR signaling since inflammatory signaling pathways and mTOR signaling are interconnected, and a feedback loop exists where inflammation, particularly increased concentration of interleukin‐1β, can activate the mTOR pathway, and in turn, hyperactive mTOR signaling can promote neuroinflammation. By suppressing chronic neuroinflammation, this feedback loop can be disrupted, leading to a reduction in mTOR activity (Zhao et al. 2023).

### 4.3 hiPSC‐NSC‐EVs Treatment in 5xFAD Mice Improved Neurogenesis and BDNF Signaling in the Hippocampus

A substantial decrease in hippocampal neurogenesis occurs in AD patients before the classical AD pathological hallmarks become apparent, a phenomenon also observed in AD models, including 5xFAD mice (Geigenmüller et al. 2024). Interestingly, decreased hippocampal neurogenesis in AD patients and models is associated with reduced hippocampal BDNF levels (Choi et al. 2018; Gao et al. 2022). An earlier study suggested that transplanting NSCs can enhance BDNF levels in an AD model (Blurton‐Jones et al. 2009), consistent with the current study demonstrating that IN administration of NSC‐derived EVs can enhance hippocampal BDNF concentration in AD brain. Using dual approaches, comprising quantification of net neurogenesis over a specific duration (i.e., the extent of mature neurons co‐expressing BrdU and NeuN in the SGZ‐GCL that are born during the BrdU injection period) and the status of ongoing neurogenesis at the time of euthanasia (i.e., the number of immature DCX+ newly born neurons in the SGZ‐GCL), the study found significantly decreased hippocampal neurogenesis in both male and female 5xFAD mice receiving Veh, which was linked with waned BDNF‐ERK‐CREB signaling. Remarkably, in both male and female 5xFAD mice, hiPSC‐NSC‐EVs treatment at 3 months of age maintained higher levels of hippocampal neurogenesis and restored BDNF, pERK, and pCREB concentrations to or near naïve control levels at 5 months of age. Higher levels of hippocampal neurogenesis observed in 5xFAD mice receiving hiPSC‐NSC‐EVs treatment are consistent with our previous study demonstrating that hiPSC‐NSC‐EVs treatment can enhance neurogenesis in naïve adult rats and in mice exhibiting peripheral inflammation‐induced neuroinflammation (Upadhya et al. 2020; Ayyubova et al. 2023).

The mechanisms underlying the maintenance of hippocampal neurogenesis and BDNF signaling at naïve control levels in 5xFAD mice are likely due to several factors. First, the hiPSC‐NSC‐EVs employed in this study are enriched with agrin (Upadhya et al. 2020), an extracellular matrix protein that enhances hippocampal neurogenesis. A previous study has shown that agrin enhances hippocampal neurogenesis by acting on low‐density lipoprotein receptor‐related protein 4 (Lrp4) expressed on NSCs, and genetic deletion of the agrin gene in excitatory neurons decreased NSC proliferation, maturation of newly born neurons, and induced hippocampus‐dependent spatial memory impairment (Zhang et al. 2019). Agrin has also been shown to induce CREB phosphorylation in hippocampal neurons (Ji et al. 1998), which can enhance BDNF concentration, as pCREB binds to the BDNF promoter, activating its transcription, resulting in increased BDNF gene and protein expression (Wang et al. 2018). Such increased BDNF signaling, mediated by agrin, can also promote better hippocampal neurogenesis in 5xFAD mice (Salta et al. 2023; Choi et al. 2018). Second, the hiPSC‐NSC‐EVs are also enriched with pentraxin 3 (PTX3), an acute‐phase protein that has been shown to promote neurogenesis via NSC proliferation in models of traumatic brain injury and stroke (Rodriguez‐Grande et al. 2015; Zhou et al. 2020). Third, diminished chronic neuroinflammation mediated by hiPSC‐NSC‐EVs in 5xFAD mice, as reported in our recent study (Madhu et al. 2024), has likely also played a role in maintaining hippocampal neurogenesis at higher levels, as neuroinflammation characterized by increased concentration of proinflammatory cytokines such as interleukin‐1 beta (IL‐1β) and IL‐6 can reduce NSC proliferation, induce apoptosis of NSCs, and decrease the survival of newly born neurons (Ekdahl et al. 2003; Chesnokova et al. 2016; Wu and Zhang 2023). Proinflammatory cytokines can also decrease BDNF signaling by increasing the methylation of the BDNF gene, which reduces its binding to CREB and subsequently decreases BDNF production (Lima Giacobbo et al. 2019). Thus, a combination of effects mediated by hiPSC‐NSC‐EVs, including the delivery of agrin and PTX3, as well as reduced chronic neuroinflammation, was among the mechanisms underlying the maintenance of higher levels of hippocampal neurogenesis associated with increased BDNF signaling in 5xFAD mice receiving hiPSC‐NSC‐EVs treatment.

### 4.4 Sex‐Specific Effects of hiPSC‐NSC‐EVs

The administration of hiPSC‐NSC‐EVs conferred a range of therapeutic benefits in both male and female 5xFAD mice. While many of the benefits were consistent across genders, some differences emerged for specific markers. Notably, the treatment with hiPSC‐NSC‐EVs exhibited a differential impact in female 5xFAD mice in a few key areas. Particularly, restoration of protein levels linked to mitophagy (PINK1, PARKIN), mTOR signaling (p‐mTOR, pS6), autophagy (ULK1, p62), and BDNF signaling (p‐ERK1/2) were more prominent in female 5xFAD mice receiving EVs than male 5xFAD mice receiving EVs. In contrast, in males, the hiPSC‐NSC‐EVs treatment had a greater impact on restoring Cox4i2 gene expression, pan‐mTOR and p‐mTOR concentration, and enhancing the production of DCX+ newly born neurons in the hippocampus. However, interactions between sex and treatment were observed only for MT‐C01, OPA1, the p‐mTOR/pan‐mTOR ratio, and ATG5, suggesting that the overall efficacy of hiPSC‐NSC‐EVs in restoring genes and proteins linked to mitochondrial health, mTOR signaling, autophagy, and neurogenesis was mostly comparable between males and females. Furthermore, levels of several proteins were higher in female naïve control mice than in male naïve control mice. These include NRF‐2, CAT, PGC‐1a, Mfn1, ATP, PINK1, PARKIN, pan‐mTOR, p‐mTOR, pS6, ATG5, LC3‐II, and p‐ERK1/2, which may suggest sexual dimorphism in metabolic regulation and higher mitochondrial function in naïve females, likely driven by hormonal factors that protect tissues from oxidative damage. Additionally, the levels of ATP, Beclin‐1, p‐mTOR/pan‐mTOR ratio, pS6, p62, and p‐ERK1/2 were higher in female 5xFAD mice receiving Veh than male 5xFAD mice receiving Veh, which may be due to higher levels of human APP and Aβ and heightened inflammation observed in female 5xFAD mice compared to male 5xFAD mice (Sil et al. 2022).

## 5 Conclusions

This study using 5xFAD mice demonstrates that IN administrations of hiPSC‐NSC‐EVs at the early stage of AD or amyloidosis can restrain the progression of key pathological changes. By targeting genes and/or proteins vital to mitochondrial integrity, balancing mTOR and autophagy, increasing BDNF signaling, and promoting hippocampal neurogenesis, this intervention provides a multifaceted approach to disease modification. These findings add to the evidence of improved hippocampus‐dependent cognitive function and significantly reduced neuroinflammation reported in our earlier study (Madhu et al. 2024) and further establish hiPSC‐NSC‐EVs as a promising therapeutic candidate. Previous studies using intravenous administration of EVs from embryonic mouse brain or human embryonic stem cell‐derived NSCs suggested their potential to enhance cognition and levels of PGC1α, FIS1, NRF‐2, sirtuin 1, and synaptic proteins, reduce Aβ plaques, and promote neuroprotective and anti‐inflammatory effects in different AD models (Apodaca et al. 2021; Li et al. 2020; Krattli Jr et al. 2026; Gao et al. 2023). The present results advance this field by demonstrating the substantial impact of IN‐administered hiPSC‐NSC‐EVs in restoring multiple genes and/or proteins that support mitochondrial health and mTOR/autophagy balance in 5xFAD mice to levels comparable to naive controls, as well as enhancing hippocampal neurogenesis. Collectively, these outcomes highlight the potential of IN‐administered hiPSC‐NSC‐EVs to significantly modify the trajectory of AD pathogenesis.

## Author Contributions

Concept: A.K.S. Research design and interpretation: A.K.S. and L.N.M. Data collection and analysis: L.N.M., S.A., S.K., R.U., Y.S., S.R., P.T., S.V.G., C.H., M.K., B.S., V.V.R., and A.K.S. The first draft of the manuscript text and figures: L.N.M. and A.K.S. Finalization of manuscript text and figures: A.K.S., L.N.M., S.V.G., and S.A. All authors provided feedback and approved the final version of the manuscript.

## Funding

This work was supported by National Institute on Aging (Grants RF1AG074256 and R01AG075440).

## Conflicts of Interest

The authors declare no conflicts of interest.

## Supporting information


**Figure S1:** Intranasally administered PKH26‐labeled hiPSC‐NSC‐EVs (red particles) incorporated into NeuN+ neurons in the dentate granule cell layer (top) and the CA3 pyramidal cell layer (bottom) of the hippocampus in 5XFAD mice. Scale = 10 μm.
**Figure S2:** Intranasally administered PKH26‐labeled hiPSC‐NSC‐EVs (red particles) incorporated into microglia in the dentate gyrus and the CA3 subfield of the hippocampus in 5XFAD mice. Scale = 10 μm.
**Figure S3:** Efficacy of Intranasal administration of extracellular vesicles (EVs) from human induced pluripotent stem cell‐derived neural stem cells (hiPSC‐NSCs) on DRP1 and OPA1 protein expression. (A) Illustrates the western blot bands for DRP1, OPA1, and GAPDH proteins from naïve control, AD‐Veh, and AD‐EVs groups. The bar charts (B–E) compare the density of DRP1 and OPA1 proteins in the hippocampus of males (B, C) and females across groups (D, E). *p < 0.05; NS not significant.
**Figure S4:** Efficacy of Intranasal administration of extracellular vesicles (EVs) from human induced pluripotent stem cell‐derived neural stem cells (hiPSC‐NSCs) on pS6 protein expression in microglia. (A–I) are representative images from naïve (A–C), AD‐Veh (D–F), and AD‐EVs (G–I) groups. Bar graphs J and K compare the percentages of microglia expressing pS6 across groups in males (J) and females (K). ***p < 0.001; ****p < 0.0001; NS not significant. A–I: Scale = 10 μm.
**Figure S5:** Efficacy of Intranasal administration of extracellular vesicles (EVs) from human induced pluripotent stem cell‐derived neural stem cells (hiPSC‐NSCs) on LC3 protein expression. Panel (A) illustrates the western blot bands for LC3‐I, LC3‐II, and GAPDH proteins from naïve control, AD‐Veh, and AD‐EVs groups. The bar charts (B–G) compare the density of LC3‐I, LC3‐II, and the LC3‐II/LC3‐I ratio in the hippocampus of males (B–D) and females across groups (E–G). *p < 0.05; NS, not significant.
**Figure S6:** Uncropped original images of DRP1, OPA1 and GAPDH western blots.
**Figure S7:** Uncropped original images of LC3‐I (top) and LC3‐II (bottom) western blots.
**Figure S8:** Illustration of an area from a negative control brain tissue section processed while performing the NeuN (green) and p62 (red) dual immunofluorescence. In this section, the primary antibody for p62 was omitted in the dual immunostaining protocol for NeuN and p62, which led to no red‐colored structures within neurons, implying that p62+ structures (red dots in Figure 6K–S) found within neuronal soma in sections processed for NeuN and p62 dual immunofluorescence staining indeed represent the localization of p62. Scale = 10 μm.

## Data Availability

All data needed to evaluate the reported findings are present in the article. The original blots of all WB data provided in the manuscript are available in Supplementary Figures 6–7.
