# Supplementary material for: Human iPSC‐NSC‐Derived Extracellular Vesicles Can Alleviate Alzheimer's Disease‐Linked Impairments in Mitochondria, mTOR Signaling, Autophagy, and Hippocampal Neurogenesis
Source: Aging Cell. 2026 Jun 16;25(6):e70590. doi: 10.1111/acel.70590 (PMC13272109; doi:10.1111/acel.70590)
Supplement: Supplementary file 1 — Figure S1: Intranasally administered PKH26‐labeled hiPSC‐NSC‐EVs (red particles) incorporated into NeuN+ neurons in the dentate granule cell layer (top) and the CA3 pyramidal cell layer (bottom) of the hippocampus in 5XFAD mice. Scale = 10 μm. Figure S2: Intranasally administered PKH26‐labeled hiPSC‐NSC‐EVs (red particles) incorporated into microglia in the dentate gyrus and the CA3 subfield of the hippocampus in 5XFAD mice. Scale = 10 μm. Figure S3: Efficacy of Intranasal administration of extracellular vesicles (EVs) from human induced pluripotent stem cell‐derived neural stem cells (hiPSC‐NSCs) on DRP1 and OPA1 protein expression. (A) Illustrates the western blot bands for DRP1, OPA1, and GAPDH proteins from naïve control, AD‐Veh, and AD‐EVs groups. The bar charts (B–E) compare the density of DRP1 and OPA1 proteins in the hippocampus of males (B, C) and females across groups (D, E). *p < 0.05; NS not significant. Figure S4: Efficacy of Intranasal administration of extracellular vesicles (EVs) from human induced pluripotent stem cell‐derived neural stem cells (hiPSC‐NSCs) on pS6 protein expression in microglia. (A–I) are representative images from naïve (A–C), AD‐Veh (D–F), and AD‐EVs (G–I) groups. Bar graphs J and K compare the percentages of microglia expressing pS6 across groups in males (J) and females (K). ***p < 0.001; ****p < 0.0001; NS not significant. A–I: Scale = 10 μm. Figure S5: Efficacy of Intranasal administration of extracellular vesicles (EVs) from human induced pluripotent stem cell‐derived neural stem cells (hiPSC‐NSCs) on LC3 protein expression. Panel (A) illustrates the western blot bands for LC3‐I, LC3‐II, and GAPDH proteins from naïve control, AD‐Veh, and AD‐EVs groups. The bar charts (B–G) compare the density of LC3‐I, LC3‐II, and the LC3‐II/LC3‐I ratio in the hippocampus of males (B–D) and females across groups (E–G). *p < 0.05; NS, not significant. Figure S6: Uncropped original images of DRP1, OPA1 and GAPDH western blots. Figure S7: Uncro [file ACEL-25-e70590-s001.docx]

**Supplemental File**

**Human iPSC-NSC-derived Extracellular Vesicles Can Alleviate Alzheimer’s Disease-Linked Impairments in Mitochondria, mTOR Signaling, Autophagy, and Hippocampal Neurogenesis**

**Leelavathi N. Madhu, Sahithi Attaluri, Sanya Kotian, Raghavendra Upadhya^#^, Yogish Somayaji, Shama Rao, Prashant Tarale, Shruthi V. Ganesh, Charles Huard, Maheedhar Kodali, Bing Shuai, Vidya V. Rao, and Ashok K. Shetty***

Institute for Regenerative Medicine, Department of Cell Biology and Genetics, Texas A&M University Naresh K Vashisht College of Medicine, Bryan/College Station, Texas, USA

**1. Materials and Methods – Additional details:**

**a) Mitochondrial function Assays:**

**Succinate Dehydrogenase (SDH) assay:** SDH in hippocampal lysates converts succinate to fumarate, generating FADH2, which transfers the electron to DCPIP (a blue-colored redox dye). When an electron is accepted, it is reduced and changes color. The hippocampus lysates were reacted with a reaction mixture containing 0.05 µL of 10 mM FAD, 0.05 µL of succinate substrate, 0.50 µL of transport reagent, 0.50 µL of 20 mM DCPIP, and 48.9 µL of PBS. The change in the color per minute was measured at 600nm.

**NAD+/NADH assay:** This assay uses WST-8, a water-soluble dye, to measure NADH levels in the samples. The tissue samples were reacted with a reaction mixture containing 0.05 µL of 1X alcohol dehydrogenase (ADH) enzyme and 49.95 µL of ADH buffer. The above reaction was maintained at 37 °C for 10 min. A later detection mixture (5 µL of WST reagent + 0.5 µL mediator reagent + 44.5 µL PBS) was added to the above reaction and incubated for 60 min at 37 °C. Absorbance was read at 450nm, and NADH concentration was calculated using the NADH standard graph.

**ATP assay:** This assay uses a couple of enzymes that catalyze the conversion of glycerol to measure ATP levels in hippocampal lysates from study groups. First, glycerol kinase converted glycerol to glycerol 3-phosphate. This step consumes the ATP present in the tissue lysates. Next, glycerol-3-phosphate oxidase catalyzes the oxidation of glycerol-3-phosphate to hydrogen peroxide. The amount of hydrogen peroxide quantified provides a direct measure of ATP levels in the sample.

**b) Western Blot (WB) Analyses:**

The total protein in the hippocampus lysate was quantified by the Pierce BCA protein assay kit (ThermoFisher Scientific), and 30 µg of protein from hippocampal lysates were loaded and separated by 4–12% NuPAGE Bis-Tris Gels (ThermoFisher Scientific). The proteins were transferred to a nitrocellulose membrane using the iBlot2 gel transfer device (ThermoFisher Scientific). The membrane was processed for protein detection using antibodies against DRP1 (1:1000; Proteintech), OPA1 (1:1000; Proteintech), and LC3 (1:1000; Proteintech). The signal was detected with the ECL detection kit (ThermoFisher) and visualized with an iBright Imaging System (ThermoFisher).

**Supplementary Figure 1**


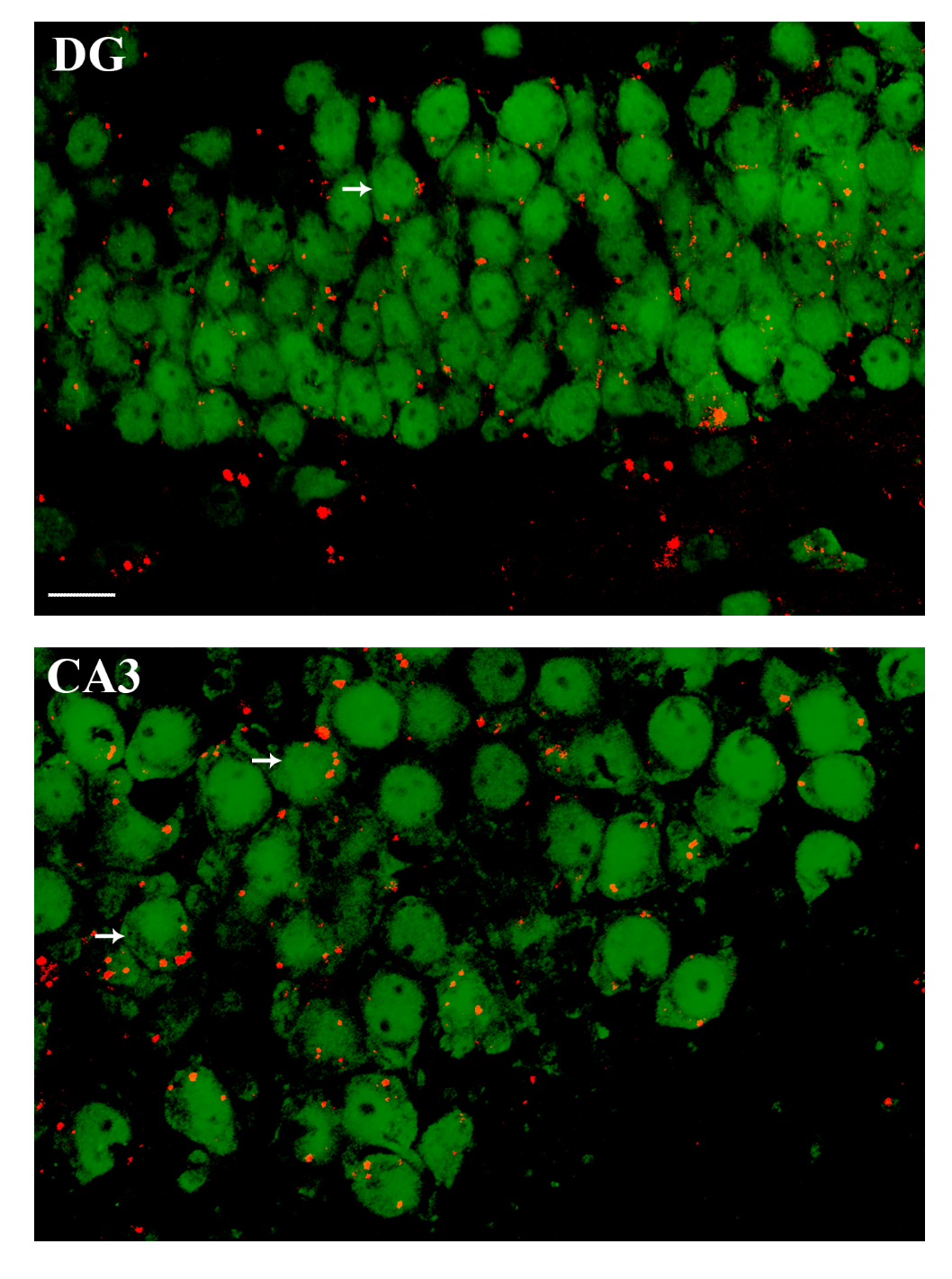


**Supplementary Figure 1:** Intranasally administered PKH26-labeled hiPSC-NSC-EVs (red particles) incorporated into NeuN+ neurons in the dentate granule cell layer (top) and the CA3 pyramidal cell layer (bottom) of the hippocampus in 5XFAD mice. Scale=10µm

**Supplementary Figure 2**


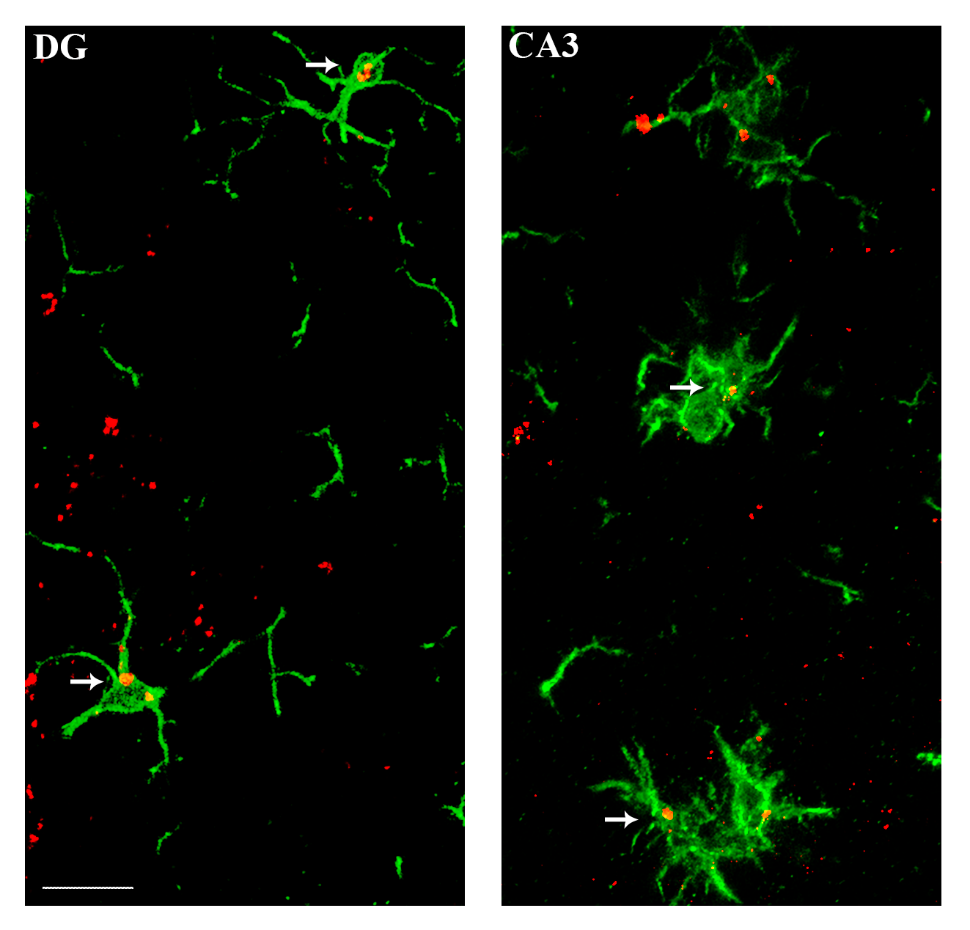


**Supplementary Figure 2:** Intranasally administered PKH26-labeled hiPSC-NSC-EVs (red particles) incorporated into microglia in the dentate gyrus and the CA3 subfield of the hippocampus in 5XFAD mice. Scale=10µm


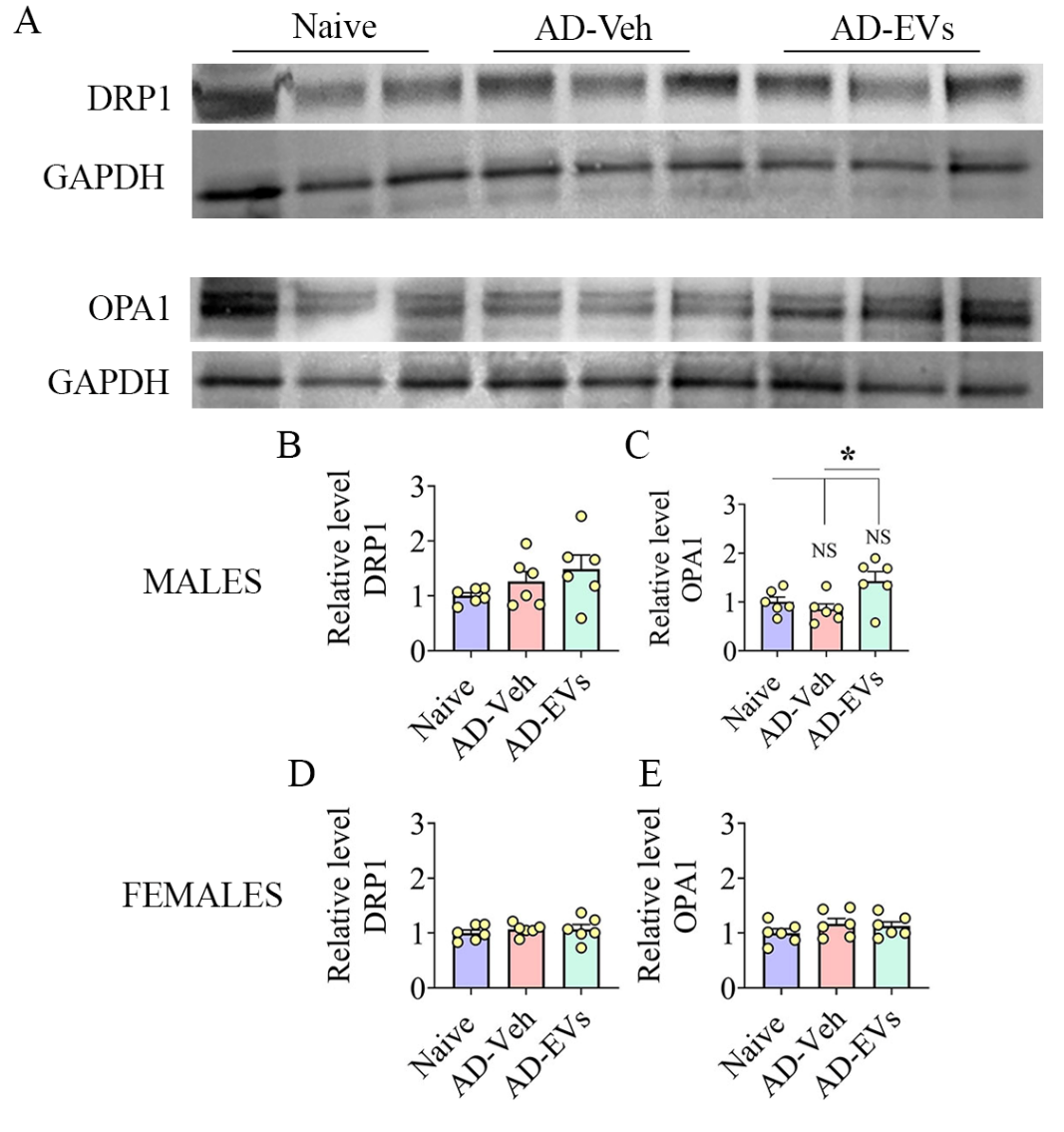
**Supplementary Figure 3**

**Supplementary Figure 3:** Efficacy of Intranasal administration of extracellular vesicles (EVs) from human induced pluripotent stem cell-derived neural stem cells (hiPSC-NSCs) on DRP1 and OPA1 protein expression. Figure A illustrates the western blot bands for DRP1, OPA1, and GAPDH proteins from naïve control, AD-Veh, and AD-EVs groups. The bar charts B–E compare the density of DRP1 and OPA1 proteins in the hippocampus of males (B-C) and females across groups (D-E). *, p < 0.05; NS not significant.

**Supplementary Figure 4**


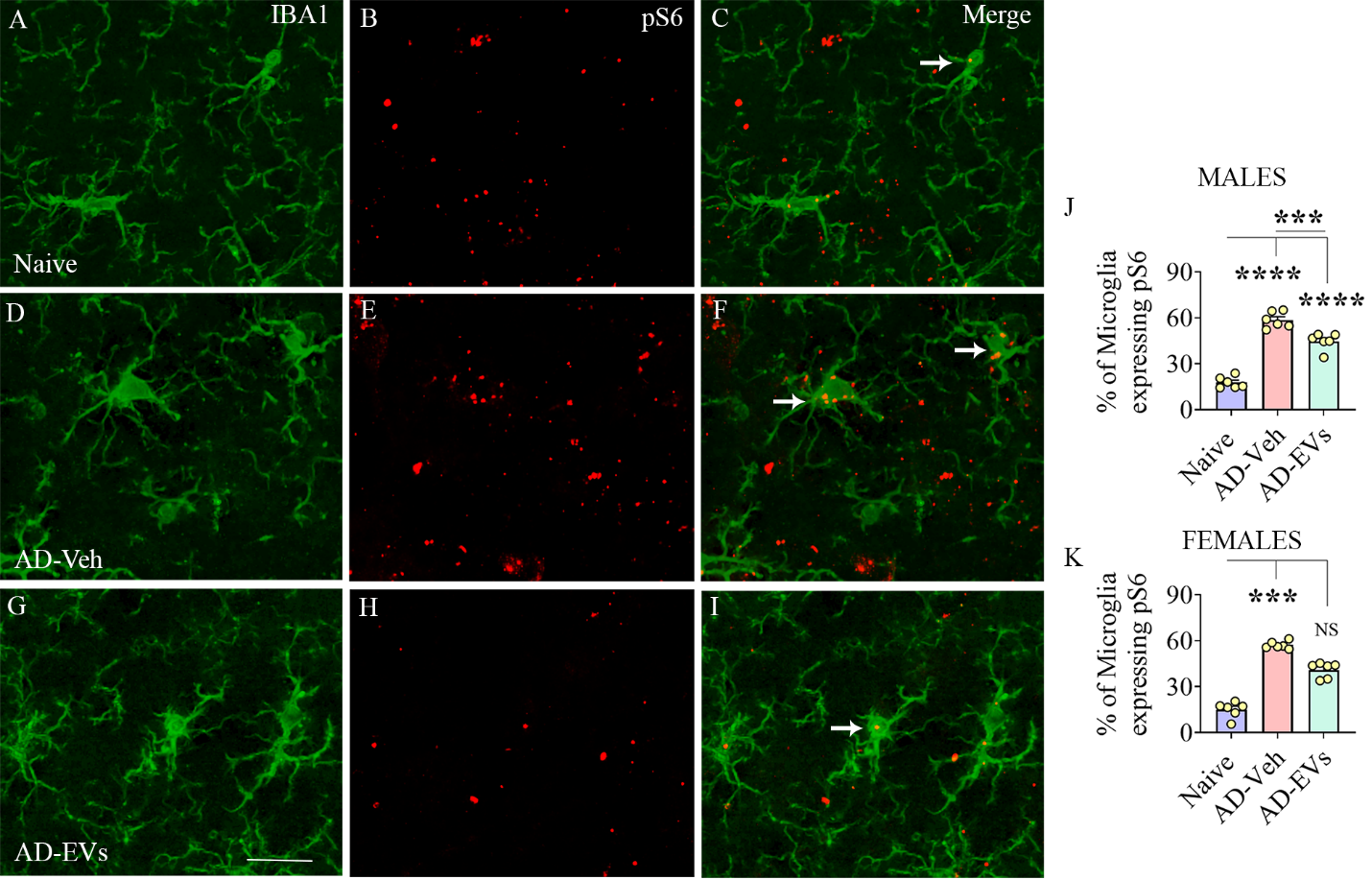


**Supplementary Figure 4:** Efficacy of Intranasal administration of extracellular vesicles (EVs) from human induced pluripotent stem cell-derived neural stem cells (hiPSC-NSCs) on pS6 protein expression in microglia. A-I are representative images from naïve (A-C), AD-Veh (D-F), and AD-EVs (G-I) groups. Bar graphs J and K compare the percentages of microglia expressing pS6 across groups in males (J) and females (K).  ***, p < 0.001; ****, p < 0.0001; NS not significant. A-I: Scale=10µm


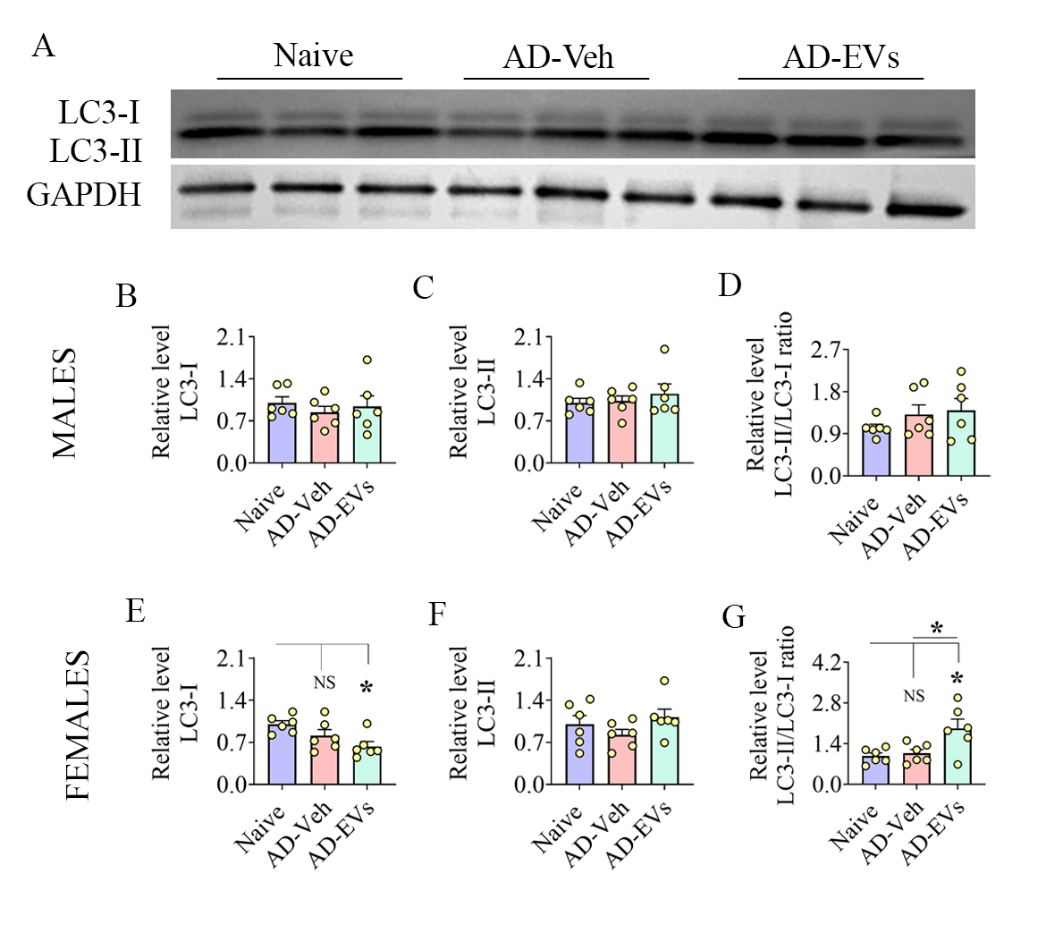
**Supplementary Figure 5**

**Supplementary Figure 5:** Efficacy of Intranasal administration of extracellular vesicles (EVs) from human induced pluripotent stem cell-derived neural stem cells (hiPSC-NSCs) on LC3 protein expression. Panel A illustrates the western blot bands for LC3-I, LC3-II, and GAPDH proteins from naïve control, AD-Veh, and AD-EVs groups. The bar charts B–G compare the density of LC3-I, LC3-II, and the LC3-II/ LC3-I ratio in the hippocampus of males (B-D) and females across groups (E-G). *, p < 0.05; NS, not significant.

**Supplementary Figure 6**


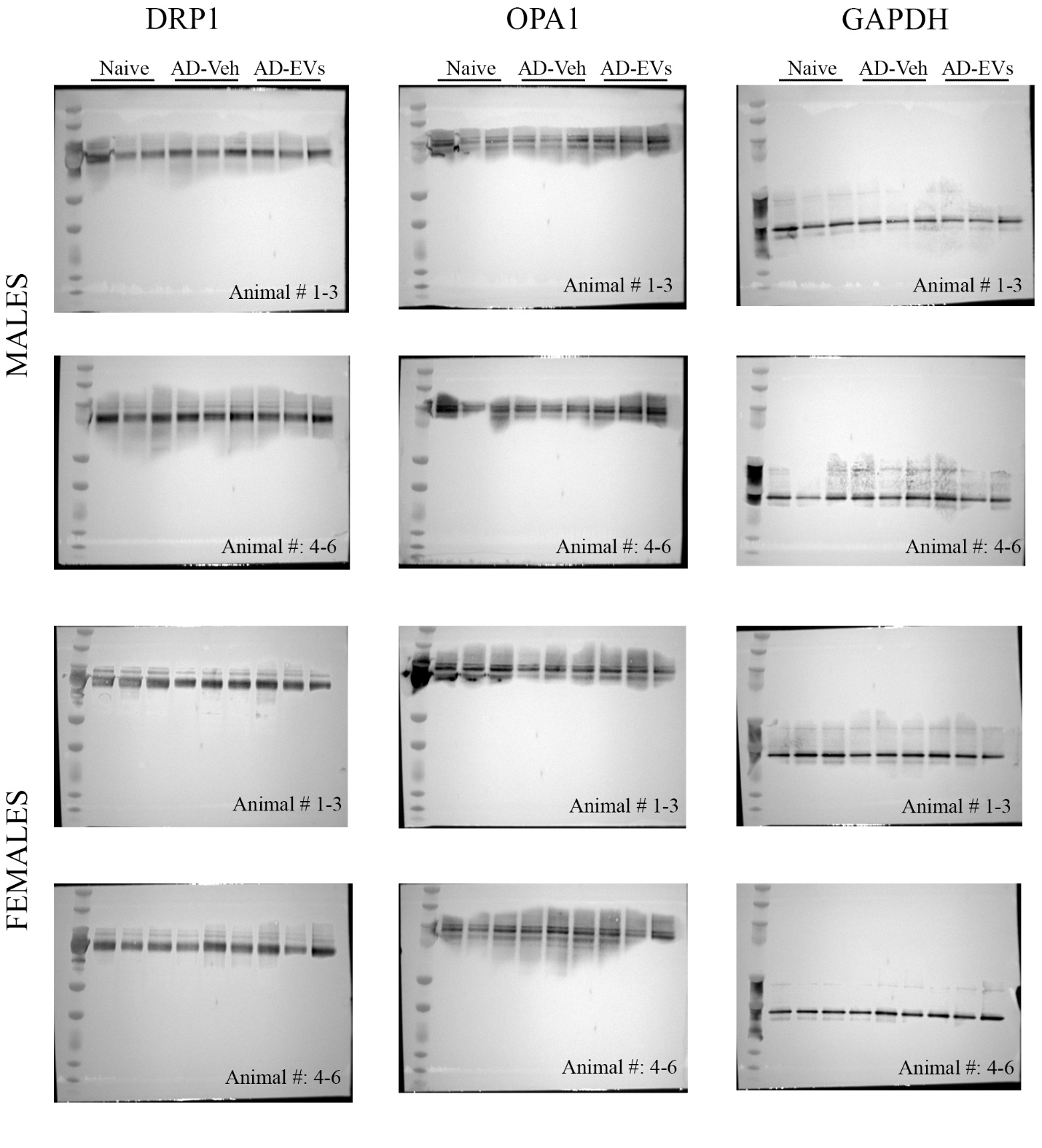


**Supplementary Figure 6:** Uncropped original images of DRP1, OPA1 and GAPDH western blots.

**Supplementary Figure 7**


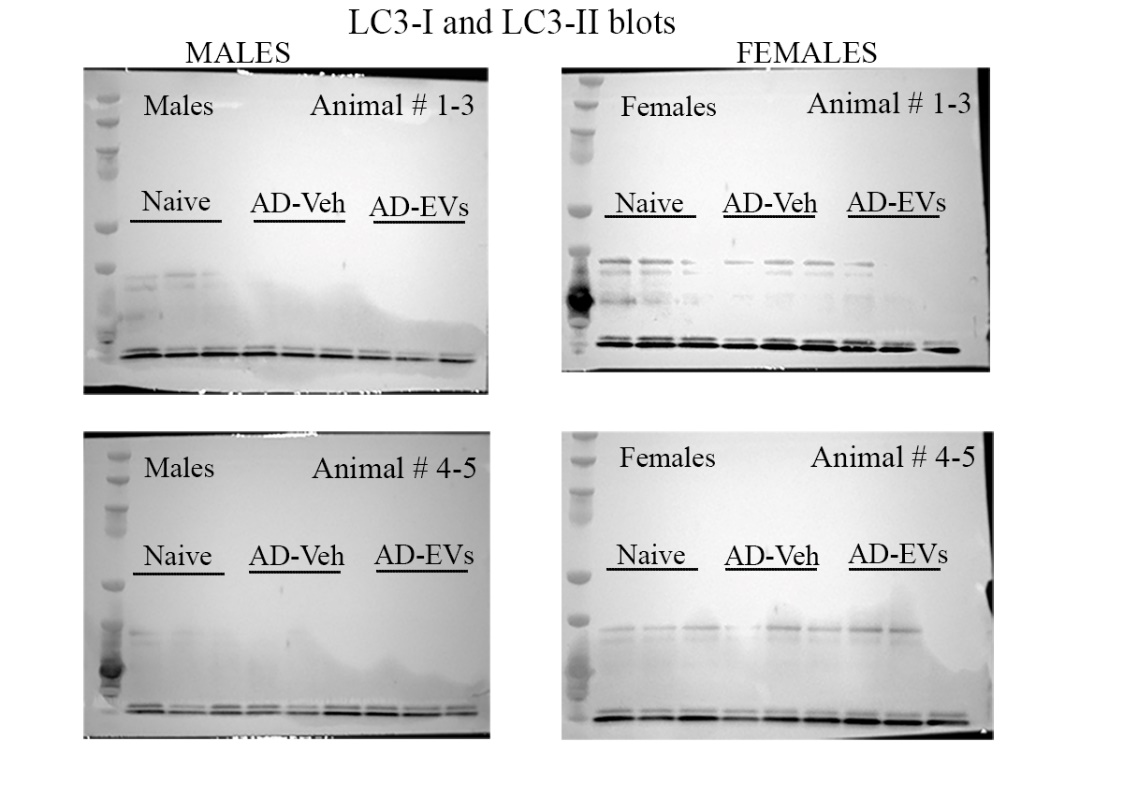


**Supplementary Figure 7:** Uncropped original images of LC3-I (top) and LC3-II (bottom) western blots.


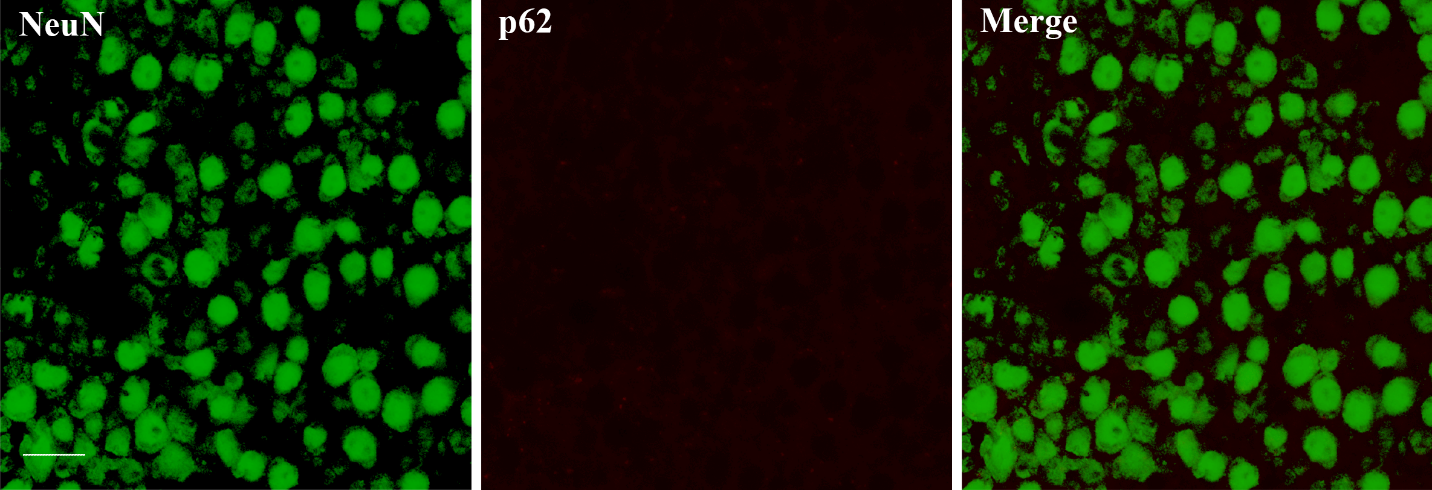
**Supplementary Figure 8**

**Supplementary Figure 8:** Illustration of an area from a negative control brain tissue section processed while performing the NeuN (green) and p62 (red) dual immunofluorescence.  In this section, the primary antibody for p62 was omitted in the dual immunostaining protocol for NeuN and p62, which led to no red-colored structures within neurons, implying that p62+ structures (red dots in Figure 6 [K-S]) found within neuronal soma in sections processed for NeuN and p62 dual immunofluorescence staining indeed represent the localization of p62. Scale=10µm
